# Multiple pregnancy with complete hydatidiform mole and coexisting normal fetus: systematic review and meta‐analysis of clinical outcomes from non‐randomized studies

**DOI:** 10.1002/uog.70104

**Published:** 2025-10-09

**Authors:** N. Salmeri, A. Pizzetti, E. Grassi, R. Cioffi, G. Mangili, M. Seckl, A. Sotiriadis, M. Candiani, P. I. Cavoretto

**Affiliations:** ^1^ Obstetrics and Gynecology Unit IRCCS San Raffaele Scientific Institute Milan Italy; ^2^ School of Medicine Vita‐Salute San Raffaele University Milan Italy; ^3^ Department of Clinical Sciences and Community Health Università degli Studi di Milano Milan Italy; ^4^ Gestational Trophoblastic Disease Centre Charing Cross Hospital Campus of Imperial College London London UK; ^5^ Second Department of Obstetrics and Gynecology, Faculty of Medicine, School of Health Sciences Aristotle University of Thessaloniki Thessaloniki Greece

**Keywords:** complete hydatidiform mole, gestational trophoblastic neoplasia, meta‐analysis, molar pregnancy, oncological outcome, pre‐eclampsia, pregnancy outcome, preterm birth, twin pregnancy

## Abstract

**Objective:**

Complete hydatidiform mole and coexisting normal fetus (CHMCF) is a rare condition for which there is significant heterogeneity in diagnosis, counseling and management of complications. The objective of this study was to summarize the prevalence of clinical outcomes in reported cases of CHMCF.

**Methods:**

A systematic literature search was conducted in PubMed, Embase and Scopus databases from inception until 1 October 2024. Case series and cohort studies including at least three cases of histologically confirmed CHMCF were included. A random‐effects model was used for meta‐analysis of proportions and heterogeneity was estimated using Higgins' *I* ^2^ index. The Newcastle–Ottawa scale and the Joanna Briggs Institute critical appraisal checklist were used to assess study quality, while certainty of evidence was assessed using Grading of Recommendations Assessment, Development and Evaluation (GRADE) methodology. The study was registered in the PROSPERO database (CRD42023431734).

**Results:**

Quantitative synthesis included 19 studies and 417 cases of CHMCF. Diagnosis was made using ultrasound in 76.0% (95% CI, 58.5–90.6%) of cases and occurred in the first trimester in 52.7% (95% CI, 34.0–71.0%). Symptoms at diagnosis were present in 80.5% (95% CI, 66.1–92.3%) of cases, with vaginal bleeding being the most common symptom both at diagnosis and later in pregnancy. The pooled proportion of elective pregnancy termination was 48.8% (95% CI, 32.7–65.1%), with 6.2% (95% CI, 1.0–13.9%) due to maternal complications. The pooled proportion of live births was 46.5% (95% CI, 36.1–57.1%), with most being delivered by Cesarean section (71.2% (95% CI, 42.4–94.4%)). Preterm birth (< 37 weeks) occurred in 67.8% (95% CI, 44.7–88.1%) of cases, very preterm birth (< 32 weeks) in 12.4% (95% CI, 0.2–33.9%) and miscarriage (fetal death < 24 weeks) in 32.7% (95% CI, 26.1–39.6%). Pre‐eclampsia was present in 17.8% (95% CI, 5.9–32.7%) of cases and postpartum hemorrhage occurred in 42.7% (95% CI, 5.1–84.8%). A small‐for‐gestational‐age neonate (birth weight < 10^th^ percentile) was delivered in 40.6% (95% CI, 12.9–70.8%) of cases. Rates of neonatal and maternal mortality were negligible. The pooled proportion of gestational trophoblastic neoplasia was 33.8% (95% CI, 25.6–42.5%); among elective terminations, continued pregnancies and live births, the rates were 14.1% (95% CI, 5.4–24.9%), 20.3% (95% CI, 12.0–29.9%) and 5.9% (95% CI, 1.9–11.2%), respectively. The evidence level according to GRADE was low to very low.

**Conclusions:**

Pregnancies with CHMCF present a high risk of maternal, obstetric and neonatal complications, including miscarriage, pre‐eclampsia, small‐for‐gestational age, postpartum hemorrhage and preterm birth. The risk of developing gestational trophoblastic neoplasia was not clearly mitigated by early pregnancy termination. Early diagnosis, referral to a maternal–fetal medicine unit with expertise in trophoblastic disorders and extensive implementation of screening protocols for preterm birth and pre‐eclampsia are recommended to facilitate timely intervention aimed at outcome improvement. © 2025 The Author(s). *Ultrasound in Obstetrics & Gynecology* published by John Wiley & Sons Ltd on behalf of International Society of Ultrasound in Obstetrics and Gynecology.

## INTRODUCTION

Twin pregnancy with complete hydatidiform mole and coexisting fetus (CHMCF) is an exceptionally rare and complex obstetric phenomenon, occurring in 1 in 100 000 pregnancies^1^. From a molecular standpoint, complete hydatidiform mole (CHM) originates from a fertilization anomaly, leading to proliferation of trophoblastic tissue without maternal DNA^2^. CHMCF typically results from fertilization of an oocyte by two sperm cells, one contributing to fetal development and the other to molar tissue formation, although cases involving a single spermatozoon with paternal genome duplication followed by abnormal cell division have also been described^3^. The resulting pregnancy includes both a developing fetus and a CHM^4^.

In singleton pregnancy, CHM is suspected on ultrasound examination based on a spectrum of findings that evolve with gestational age, from an empty gestational sac at 4–5 weeks, to a polypoid intrauterine mass at 6–7 weeks and, by 8 weeks, to villous hydrops presenting as a multicystic, hypervascular ‘snowstorm’ mass without fetal tissue, typically accompanied by markedly elevated human chorionic gonadotropin (hCG) levels^5^, ^6^, ^7^. Elevated hCG levels, coupled with hypervascular and disrupted placentation, are commonly associated with vaginal bleeding, hyperemesis gravidarum and symptoms of hyperthyroidism^8^. Clinical and sonographic suspicion of a CHM pregnancy leads to pregnancy termination, followed by histopathological confirmation of the diagnosis. Long‐term follow‐up, including serial hCG measurements, in accordance with International Federation of Gynecology and Obstetrics (FIGO) guidelines^9^, is crucial for detecting and managing potential oncological complications, with persistent trophoblastic disease or postmolar gestational trophoblastic neoplasia (GTN) manifesting in 15–20% of cases^8^.

The natural history of CHMCF remains poorly understood, encompassing a wide range of potential fetal and neonatal outcomes^9^. Additionally, the presence of molar tissue may induce a spectrum of maternal complications, such as hyperemesis gravidarum, gestational hypertension and pre‐eclampsia. In rare cases, it may also lead to GTN^10^, ^11^, ^12^.

Owing to its rarity, the prevalence of obstetric outcomes in CHMCF and prognostic factors for pregnancy evolution are poorly defined, hence the absence of standardized clinical guidelines. The management of CHMCF pregnancies thus remains a challenge^12^.

The objective of this systematic review and meta‐analysis was to summarize and quantify the prevalence of clinical outcomes in reported cases of CHMCF, categorizing data based on relevant clinical features that may aid in the prognostic evaluation of patients with CHMCF.

## METHODS

### Study design

This systematic review and meta‐analysis was conducted in accordance with the Preferred Reporting Items for Systematic Reviews and Meta‐Analyses (PRISMA) guidelines^13^. The study protocol was registered prospectively in the publicly accessible PROSPERO database (ref.: CRD42023431734). Approval by the local ethics committee was not deemed to be necessary for this study, given that data were obtained from published literature.

### Search strategy

A systematic search was conducted in PubMed, Embase and Scopus databases from the inception of each database until 1 October 2024, using a combination of three queries in PubMed and equivalent strategies in the other databases: (‘molar pregnancy’ AND ‘viable fetus’ OR ‘hydatidiform mole’ AND ‘coexisting fetus’ OR ‘hydatiform mole’ AND ‘coexisting fetus’), ‘complete mole’ and (‘obstetric outcome’ OR ‘complications’ OR ‘management’ OR ‘perinatal outcome’). Detailed search strategies for all databases are provided in Appendix S1. The reference lists of relevant papers were examined manually to identify any relevant articles not captured by the electronic searches. Duplicate articles were excluded.

The search was limited to human studies published since 1980, with no restrictions for geographic location. Only full‐length manuscripts written in English and published in peer‐reviewed journals were included.

Case series and cohort studies, including at least three cases of histologically confirmed CHMCF, were included. Isolated case reports were excluded, as were abstracts and studies that did not report original results (reviews, editorials and comments). When multiple studies reported cases from the same cohort, only the larger and more recent study was retained in the pooled estimate to avoid data redundancy.

Screening and assessment of article eligibility were performed independently by two authors (A.P., E.G.). Disagreements were resolved by discussion with a third author (N.S.).

### Data extraction

Raw data from original studies were extracted by two authors independently (A.P., E.G.). If the published data were insufficient to evaluate the prespecified outcomes, we contacted the corresponding author (P.I.C.) to request the complete dataset or detailed case‐level information necessary for analysis. The following data were collected and tabulated onto a standardized data extraction form: (i) general study characteristics, including name of the first author, year of publication, study period, study design and sample size (i.e. number of CHMCF cases with a histological diagnosis); (ii) demographic characteristics of study population, including age, obstetric history and mode of conception (natural or assisted reproductive technology (ART)); (iii) molar pregnancy characteristics, including gestational age at diagnosis, method of diagnosis, clinical and radiological signs and symptoms, and pregnancy management after the diagnosis (termination of pregnancy (TOP), with timing and reason for termination, or continuation of pregnancy); (iv) molar pregnancy outcomes, including maternal complications, neonatal outcomes, method of delivery, gestational age at delivery, birth weight (with corresponding percentile calculated using the Fetal Medicine Foundation calculator^14^) and oncological outcome at follow‐up (number of cases with GTN, and definition or diagnostic criteria adopted for GTN).

### Quality assessment

Two authors (A.P., E.G.) independently assessed the quality of the included studies. Disagreements were resolved by discussion with a third author (N.S.).

The criteria proposed in the Newcastle–Ottawa scale (NOS) for non‐randomized studies were used to assess the quality of cohort studies^15^. According to this scoring system, eight items were considered for each article to evaluate selection, comparability, and reporting of outcomes, giving a score out of nine. As a result, studies were rated as high quality (6–9 points), fair quality (3–5 points) or poor quality (0–2 points).

The Joanna Briggs Institute (JBI) critical appraisal checklist was used to evaluate the quality of case series^16^. For each case series, a checklist of 10 items was completed; some items relate to risk of bias, while others relate to ensuring adequate reporting and statistical analysis. Considering that this tool emphasizes the importance of consecutive and complete inclusion of participants (items 4 and 5, respectively), any article that did not report consecutive and complete inclusion of cases was considered as a low‐quality series.

The certainty of the evidence for the estimates of clinical outcomes was assessed using the Grading of Recommendations Assessment, Development and Evaluation (GRADE) approach^17^.

### Data synthesis

The pooled proportions of the following predefined obstetric outcomes in the CHMCF population were calculated when at least two studies were available: (i) diagnosis: symptoms at diagnosis, specifically vaginal bleeding, hyperemesis gravidarum and hypertensive disorders, diagnosis suspected on ultrasound evaluation and first‐trimester diagnosis; (ii) pregnancy course: elective TOP and its indication (maternal request or maternal complication), continuation of pregnancy, spontaneous TOP, either miscarriage (< 24 weeks' gestation) or intrauterine fetal death (IUFD) (≥ 24 weeks' gestation), and maternal complications during pregnancy, including hypertensive disease (any or pre‐eclampsia), hyperthyroidism, hyperemesis gravidarum and vaginal bleeding; (iii) delivery outcomes: live birth; gestational age at delivery (term birth ≥ 37 weeks, preterm birth < 37 weeks or very preterm birth < 32 weeks), mode of delivery, including Cesarean section or vaginal birth and iatrogenic delivery for maternal complications, neonatal complications (including small‐for‐gestational age (SGA) defined as birth weight < 10^th^ percentile) and neonatal mortality, and maternal peripartum complications, including postpartum hemorrhage and maternal mortality; (iv) prevalence of GTN, stratified by clinical subgroup to account for the influence of pregnancy management (elective TOP, continued pregnancy or live birth).

We employed a random‐effects model for meta‐analysis of proportions, using the command ‘metaprop’^18^ in STATA version 18 (StataCorp LLC., College Station, TX, USA) to calculate 95% CIs using the exact binomial method^19^. To stabilize variances and normalize the distributions of proportions extracted from the included studies, the Freeman–Tukey double arcsine transformation was applied. This method was chosen to account for intrastudy variability using the binomial distribution, handling proportions near 0 or 1 while avoiding the direct issues associated with logit transformation in such cases. The aggregate estimates obtained were back‐transformed to their original format of proportions for interpretation. In cases of low heterogeneity and when many original studies reported a proportion of zero for a particular outcome, raw proportions with fixed‐effects models were used to prevent distortions in the pooled estimates. Results were reported as overall percentage estimates with 95% CI. The heterogeneity of the pooled estimates was assessed using Higgins' *I* ^2^ index, which was interpreted as follows: 0–24%, insignificant heterogeneity; 25–49%, low heterogeneity; 50–74%, moderate heterogeneity; and ≥ 75%, high heterogeneity^20^. Pooled prevalences for GTN were compared between subgroups using a two‐sided *Z*‐test for proportions. *P* < 0.05 was used to indicate statistical significance.

## RESULTS

**Figure 1:**
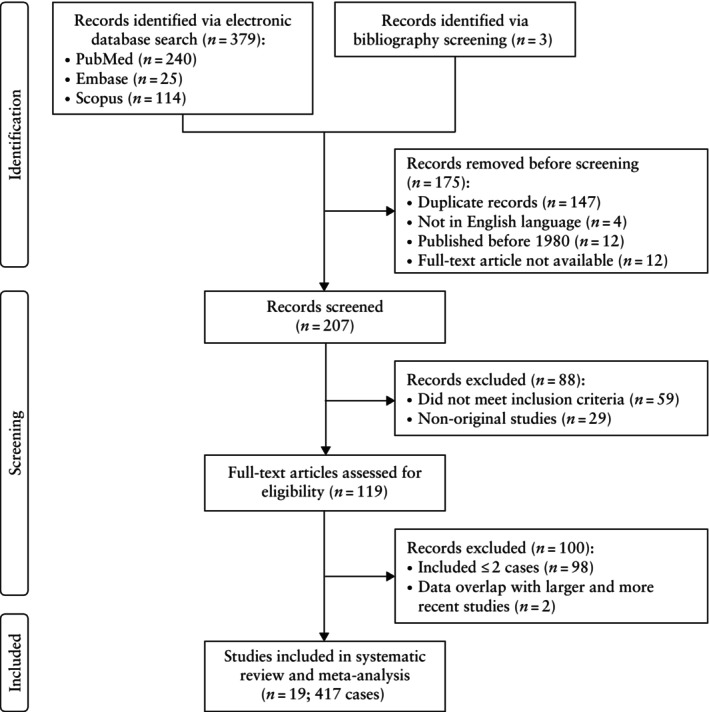
PRISMA flowchart summarizing inclusion of studies on histologically confirmed cases of complete hydatidiform mole and coexisting normal fetus in systematic review and meta‐analysis.

A total of 379 articles were identified via the database search, with three additional records identified through bibliography screening (Figure 1). After removing duplicate records (*n* = 147), articles not in English (*n* = 4), articles published before 1980 (*n* = 12) and articles for which the full text was not available (*n* = 12), 207 records were screened and 119 full‐text articles were assessed for eligibility. Of those, 21 articles were identified as eligible. However, two articles were excluded to avoid data redundancy because they contained cases that were reported in a subsequent larger publication^1^. Ultimately, 19 articles were available for quantitative synthesis, comprising 417 cases of CHMCF^1^, ^10^, ^21^, ^22^, ^23^, ^24^, ^25^, ^26^, ^27^, ^28^, ^29^, ^30^, ^31^, ^32^, ^33^, ^34^, ^35^, ^36^, ^37^.

The characteristics of the included studies are summarized in Table 1. Of the 19 included studies, 12 were case series and seven were cohort studies. The mean number of cases per study was 21.9, although significant between‐study heterogeneity in population size was observed (range, 3–141). Fifteen studies (193 cases) reported the maternal age of patients diagnosed with CHMCF: all women were between 18 and 41 years old, with none at the extremes of reproductive age. Thirteen studies reported on the mode of conception, of which eight included both patients who conceived spontaneously and those who conceived by ART. As per the inclusion criteria, the definition of cases was consistent among the included studies, with all reporting histopathological confirmation of the diagnosis.

**Table 1:** Characteristics of 19 studies on histologically confirmed cases of complete hydatidiform mole and coexisting normal fetus (CHMCF) included in systematic review and meta‐analysis

Study	Country	Study design	Study period	Cases (*n*)	Continued pregnancies (*n* (%))	Mode of conception (*n*/*N*)	Definition of cases (CHMCF)	Definition of GTN
Fishman (1998)^21^	USA	Retro cohort	1966–1997	7	7 (100)	Spontaneous (7/7)	Twin pregnancy comprising complete mole and coexisting fetus; histopathological confirmation	Persistent GTN after delivery or uterine evacuation*
Giorgione (2017)^22^	Italy	Case series	2000–2013	13	11 (84.6)	Spontaneous (9/13), ART (4/13)	Twin pregnancy with normal fetus and placenta in addition to hydatidiform mole; confirmation by two referral pathologists	FIGO criteria^9^
Hajri (2024)^1^	France	Retro cohort	2001–2022	141	108 (76.6)	Spontaneous (110/141), ART (31/141)	Complete mole associated with ≥ 1 fetus with normal referral sonography and no indication for CVS or amniocentesis; histopathological confirmation	FIGO criteria^9^
Hancock (2006)^23^	UK	Retro cohort	1986–2004	9	9 (100)	NS	Clinical or histopathological suspicion of twin molar gestation; histopathology review with immunohistochemical p57kip2 staining	Persistent GTN after delivery or uterine evacuation
Hemida (2022)^24^	Egypt	Case series	2015–2020	6	4 (66.7)	Spontaneous (5/6), ART (1/6)	Molar pregnancy with coexisting live fetus diagnosed by clinical, ultrasound and serum hCG criteria; histopathological confirmation	Persistent GTN after delivery or uterine evacuation
Jauniaux (1997)^25^	UK	Case series	NS	4	3 (75.0)	Spontaneous (4/4)	Clinical suspicion of CHMCF; histopathological confirmation	Postmolar GTN
Kihara (2012)^26^	Japan	Case series	1991–2011	17	15 (88.2)	NS	Clinical suspicion of CHMCF; histopathological confirmation; DNA polymorphism analysis and/or p57kip2 immunohistochemistry	Postmolar GTN
Kutuk (2014)^27^	Turkey	Case series	2007–2012	7	5 (71.4)	Spontaneous (5/7), ART (2/7)	Vacuolar placenta adjacent to normal‐appearing placenta; fetus without major congenital malformation or severe growth restriction; normal fetal karyotype; histopathological confirmation	Persistent GTN after delivery or uterine evacuation
Lee (2010)^28^	South Korea	Case series	1998–2008	6	5 (83.3)	Spontaneous (2/6), ART (4/6)	Clinical suspicion of CHMCF; histopathological confirmation	Persistent GTN after delivery or uterine evacuation
Liang (2022)^29^	China	Retro cohort	1990–2020	11	2 (18.2)	NS	Clinical suspicion of CHMCF; histopathological confirmation	FIGO criteria^9^
Lin (2017)^30^	USA, Brazil	Retro cohort, multicenter	1966–2015	72	53 (73.6)	Spontaneous (63/72), ART (9/72)	Clinical suspicion of CHMCF; histopathological confirmation; cytogenetic or ploidy analysis in case of uncertain histological diagnosis	FIGO criteria^9^
Lu (2022)^31^	China	Case series	2004–2021	15	1 (6.7)	Spontaneous (14/15), ART (1/15)	Clinical suspicion of CHMCF; confirmation by immunohistochemistry and DNA genotyping	FIGO criteria^9^
Marcorelles (2005)^32^	France	Case series	NS	4	3 (75.0)	Spontaneous (4/4)	Complete hydatidiform mole with coexisting live con‐twin fetus; histopathological confirmation	Persistent GTN after delivery or uterine evacuation
McNally (2021)^33^	USA	Case series	1990–2015	5	3 (60.0)	NS	Twin pregnancy and complete mole; histopathological confirmation	Persistent GTN after delivery or uterine evacuation
Miller (1993)^34^	Canada	Case series	NS	4	4 (100)	Spontaneous (4/4)	Twin molar and normal intrauterine pregnancy; histopathological confirmation	Persistent GTN after delivery or uterine evacuation†
Niemann (2007)^35^	Denmark	Retro cohort	1986–2003	8	3 (37.5)	NS	Twin pregnancy with normal placenta connected to normal fetus and placenta with hydatidiform change; ploidy determined by karyotyping; histopathological confirmation	Persistent GTN after delivery or uterine evacuation
Sebire (2002)^36^	UK	Retro cohort	NS	77	53 (68.8)	NS	Twin pregnancy with hydatidiform mole and healthy cotwin; histopathological confirmation	Charing Cross criteria^36^
Steller (1994)^10^	USA	Case series	1965–1992	8	0 (0)	Spontaneous (8/8)	Twin pregnancy comprising hydatidiform mole and coexisting fetus; histopathological confirmation and DNA ploidy assessment	Persistent GTN after delivery or uterine evacuation‡
Zilberman Sharon (2019)^37^	Israel	Case series	2008–2018	3	1 (33.3)	Spontaneous (1/3), ART (2/3)	Clinical suspicion of CHMCF; histopathological confirmation	Persistent GTN after delivery or uterine evacuation

* Human chorionic gonadotropin (hCG) levels plateaued or increased for two or more consecutive determinations.

† hCG levels increased for two or more consecutive determinations.

‡ hCG levels plateaued or increased for at least three consecutive determinations. ART, assisted reproductive technology; CVS, chorionic villous sampling; FIGO, International Federation of Gynecology and Obstetrics; GTN, gestational trophoblastic neoplasia; NS, not stated; Retro, retrospective.

In contrast, the definition of GTN used in the included studies was heterogeneous (Table 1). Five studies adopted the FIGO 2002 criteria for GTN^9^ and one study adopted the Charing Cross criteria^36^. Eleven studies defined the oncological outcome as GTN that persisted after delivery or uterine evacuation of molar tissue, of which only three studies specified the hCG assessments undertaken. The remaining two studies used the outcome of postmolar GTN, without specifying diagnostic criteria such as hCG trends or general monitoring.

Risk‐of‐bias assessment is summarized in Table S1 for cohort studies (mean NOS score, 7.3 (range, 6–9)) and Table S2 for case series (mean number of items scored ‘yes’ on JBI checklist, 8.8 (range, 6–10)).

Pooled proportions of clinical outcomes are summarized in Table 2 and forest plots are presented in Figure S1. Symptoms at diagnosis were reported in a pooled proportion of 80.5% (95% CI, 66.1–92.3%) of CHMCF cases. Vaginal bleeding was the most common symptom, described in 61.6% (95% CI, 48.2–74.2%) of cases, with low heterogeneity (*I* ^2^ = 25.7%). According to 14 of the included studies, the pooled prevalence of cases for which a diagnosis of CHMCF was suspected on ultrasound evaluation was 76.0% (95% CI, 58.5–90.6%), albeit with high heterogeneity (*I* ^2^ = 75.1%). Diagnosis occurred during the first trimester in 52.7% (95% CI, 34.0–71.0%) of cases (*I* ^2^ = 73.1%).

**Table 2:** Pooled proportions of clinical outcomes in 417 cases of complete hydatidiform mole and coexisting normal fetus (CHMCF)

Outcome	Studies (*n*)	Pooled prevalence (95% CI) (%)	*I* ^2^ (%)
*Diagnosis of CHMCF*			
Symptoms at diagnosis			
Any	12	80.5 (66.1–92.3)	47.1
Vaginal bleeding	12	61.6 (48.2–74.2)	25.7
Hyperemesis gravidarum	9	28.0 (7.9–52.7)	71.6
Hypertensive disorder	9	2.8 (0.0–11.4)	18.3
Diagnosis suspected on US evaluation	14	76.0 (58.5–90.6)	75.1
First‐trimester diagnosis	12	52.7 (34.0–71.0)	73.1
*Pregnancy course and complications*			
Elective TOP	19	48.8 (32.7–65.1)	85.4
Maternal request	19	33.1 (17.4–50.6)	87.6
Maternal complication	19	6.2 (1.0–13.9)	67.4
Continuation of pregnancy	19	66.3 (49.0–81.9)	87.7
Spontaneous TOP*			
Miscarriage (< 24 weeks)	18	32.7 (26.1–39.6)	0.0
IUFD (≥ 24 weeks)	17	0.03 (0.01–0.05)	0.0
Maternal complications*			
Hypertensive disease (any)	15	24.3 (10.2–40.9)	71.7
Pre‐eclampsia	15	17.8 (5.9–32.7)	68.4
Hyperthyroidism	11	10.2 (2.8–20.2)	0.0
Hyperemesis gravidarum	11	18.2 (1.6–42.4)	47.7
Vaginal bleeding	11	76.2 (68.1–83.7)	0.0
*Delivery outcomes*			
Live birth*	18	46.5 (36.1–57.1)	32.6
Gestational age at delivery†			
Term birth (≥ 37 weeks)	14	32.2 (11.9–55.3)	18.6
Preterm birth (< 37 weeks)	14	67.8 (44.7–88.1)	18.6
Very preterm birth (< 32 weeks)	14	12.4 (0.2–33.9)	19.0
Mode of delivery†			
Cesarean section	11	71.2 (42.4–94.4)	0.0
Vaginal birth	11	28.8 (5.6–57.6)	0.0
Iatrogenic delivery (for maternal complications)	12	25.2 (1.6–58.4)	46.5
Neonatal complications†			
Small‐for‐gestational age‡	6	40.6 (12.9–70.8)	0.0
Neonatal mortality	15	0.04 (0.01–0.07)	0.0
Maternal peripartum complications†			
Postpartum hemorrhage	6	42.7 (5.1–84.8)	40.5
Maternal mortality	14	0.0 (0.0–0.0)	0.0
*GTN* §			
GTN (total)	18	33.8 (25.6–42.5)	43.8
GTN in elective TOP	14	14.1 (5.4–24.9)	66.7
GTN in continued pregnancy	14	20.3 (12.0–29.9)	45.9
GTN in live birth	12	5.9 (1.9–11.2)	0.0

* Proportion calculated out of number of continued pregnancies.

† Proportion calculated out of number of live births.

‡ Defined as birth weight < 10^th^ percentile.

§ Proportion calculated out of number of patients with reported follow‐up for diagnosis of gestational trophoblastic neoplasia (GTN). IUFD, intrauterine fetal death; TOP, termination of pregnancy; US, ultrasound.

All of the included studies reported on elective TOP, with pooled prevalences for all elective TOP, TOP for maternal request and TOP for maternal complications of 48.8% (95% CI, 32.7–65.1%), 33.1% (95% CI, 17.4–50.6%) and 6.2% (95% CI, 1.0–13.9%), respectively. However, heterogeneity of the estimates was moderate to high (*I* ^2^ values of 85.4%, 87.6% and 67.4%, respectively).

A pooled proportion of 66.3% (95% CI, 49.0–81.9%) of cases opted for continuation of pregnancy, also with high heterogeneity between studies (*I* ^2^ = 87.7%). Of those, 32.7% (95% CI, 26.1–39.6%) had a miscarriage before 24 weeks, whereas the pooled prevalence of IUFD was 0.03% (95% CI, 0.01–0.05%), with only four studies reporting one or more cases of IUFD in their cohort. The most common maternal complication during pregnancy was vaginal bleeding (pooled prevalence, 76.2% (95% CI, 68.1–83.7%)), with a consistent estimate reported by the included studies (*I* ^2^ = 0.0%). Pre‐eclampsia was present in a pooled proportion of 17.8% (95% CI, 5.9–32.7%) of cases, although heterogeneity was moderate (*I* ^2^ = 68.4%).

A total of 18 studies reported data on live births, which accounted for a pooled proportion of 46.5% (95% CI, 36.1–57.1%) of cases, with low heterogeneity (*I* ^2^ = 32.6%). Most live births occurred preterm (< 37 weeks' gestation) (pooled prevalence, 67.8% (95% CI, 44.7–88.1%)), with insignificant heterogeneity (*I* ^2^ = 18.6%); however, the pooled rate of very preterm birth (< 32 weeks' gestation) was only 12.4% (95% CI, 0.2–33.9%). There was a paucity of data in relation to etiology or indication for preterm birth, precluding subgroup analysis.

Most live births occurred by Cesarean section (pooled prevalence, 71.2% (95% CI, 42.4–94.4%)), with no heterogeneity of the estimate (*I* ^2^ = 0.0%). Postpartum hemorrhage was present in 42.7% (95% CI, 5.1–84.8%) of cases, with low heterogeneity (*I* ^2^ = 40.5%). The pooled proportion of maternal mortality was 0%, with one only study reporting a single case of maternal death. Regarding neonatal complications, SGA was reported in a consistent pooled proportion of 40.6% (95% CI, 12.9–70.8%; *I* ^2^ = 0.0%) of cases. Only four studies reported neonatal deaths, with rates ranging from 5% to 30%, while most of the included studies reported no case of neonatal death. A total of 11/129 neonatal deaths were recorded, with a pooled prevalence of 0.04% (95% CI, 0.01–0.07%).

**Figure 2:**
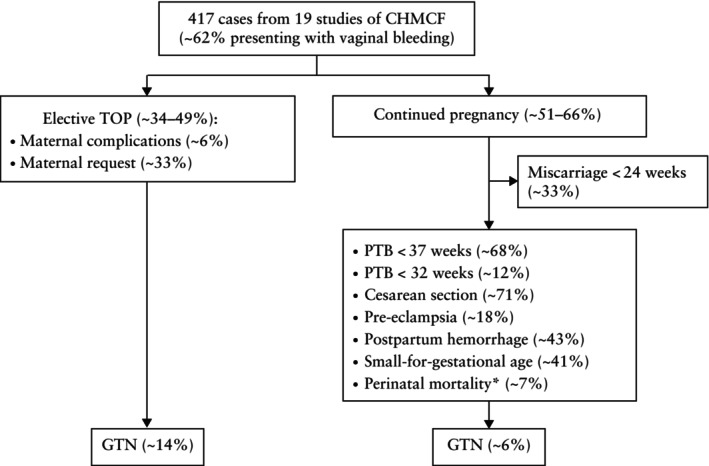
Flowchart summarizing results of meta‐analysis on complete hydatidiform mole and coexisting normal fetus (CHMCF). Uncertainty in estimates reflects large confidence intervals. Note that denominators differ between pooled analyses for elective termination of pregnancy (TOP) and continued pregnancy. *Sum of intrauterine and neonatal deaths. GTN, gestational trophoblastic neoplasia; PTB, preterm birth.

Oncological follow‐up for assessment of GTN was reported in 18 studies. The pooled proportion of GTN was 33.8% (95% CI, 25.6–42.5%), with low heterogeneity (*I* ^2^ = 43.8%). Accounting for pregnancy management, the pooled proportion of GTN was 14.1% (95% CI, 5.4–24.9%) in pregnancies that underwent elective TOP, 20.3% (95% CI, 12.0–29.9%) in continued pregnancies and 5.9% (95% CI, 1.9–11.2%) in pregnancies that led to a live birth. Comparing the rates of GTN between these groups indicated a slight benefit of TOP over continuation of pregnancy (*P* = 0.03) and of live birth over TOP (*P* = 0.009); however, the comparability of these proportions may be limited by heterogeneity in outcome definitions and study protocols. The study results are summarized in Figure 2.

Sensitivity analyses restricted to cohort studies yielded consistent results (Table S3). The certainty of evidence according to GRADE methodology was judged as low to very low (Table S4).

## DISCUSSION

### Summary of main findings

**Figure 3:**
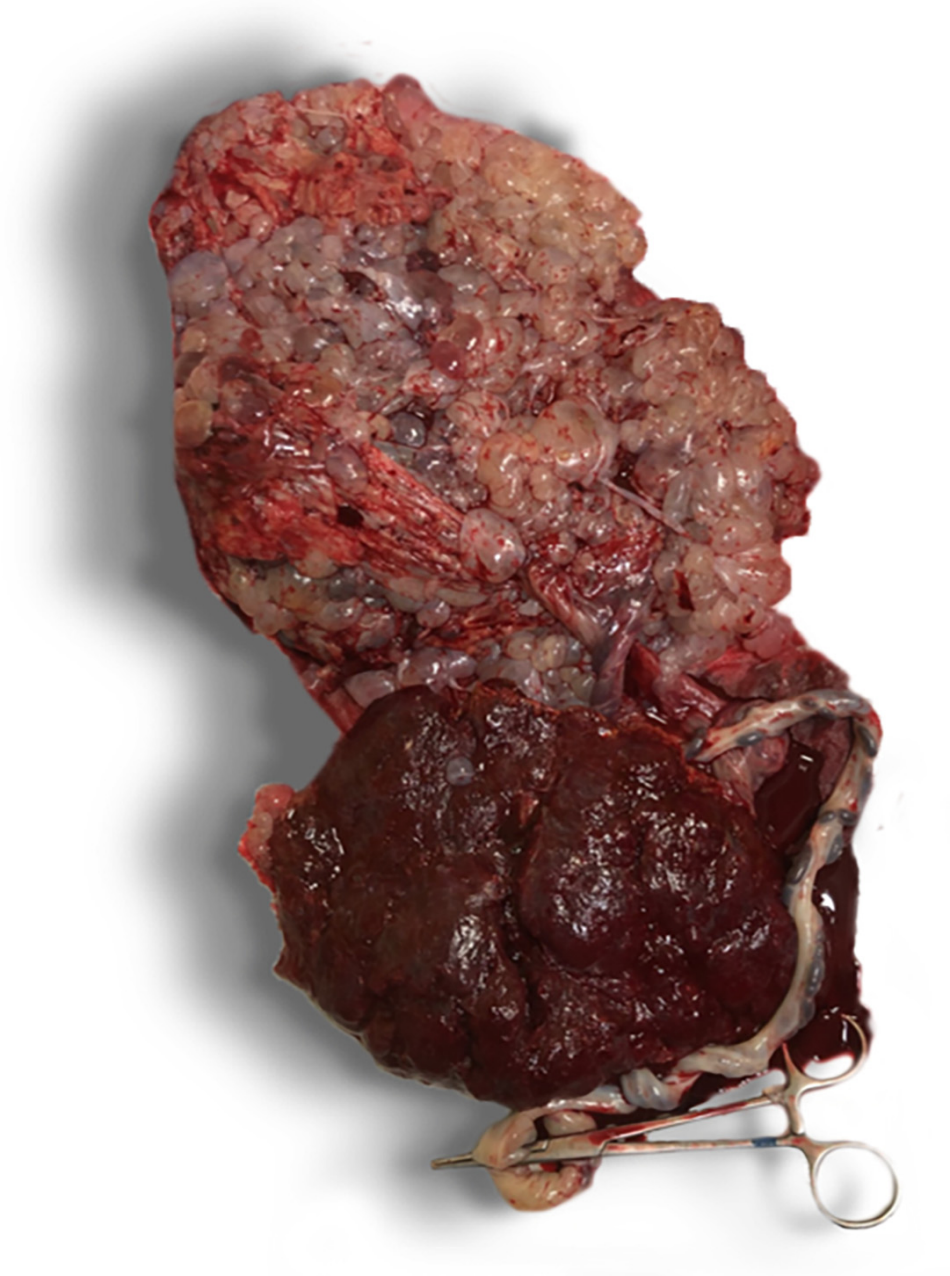
Photograph of maternal surface of placenta after birth in original case of complete hydatidiform mole and coexisting normal fetus seen at our center. Molar placenta at top appears larger and lighter in color compared with normal placenta below, which is smaller and darker in color and shows emergence of umbilical cord. Molar placenta exhibits distinct macroscopic appearance, characterized by clusters of vesicles resembling bunches of grapes, resulting from transformation of chorionic villi.

**Figure 4:**
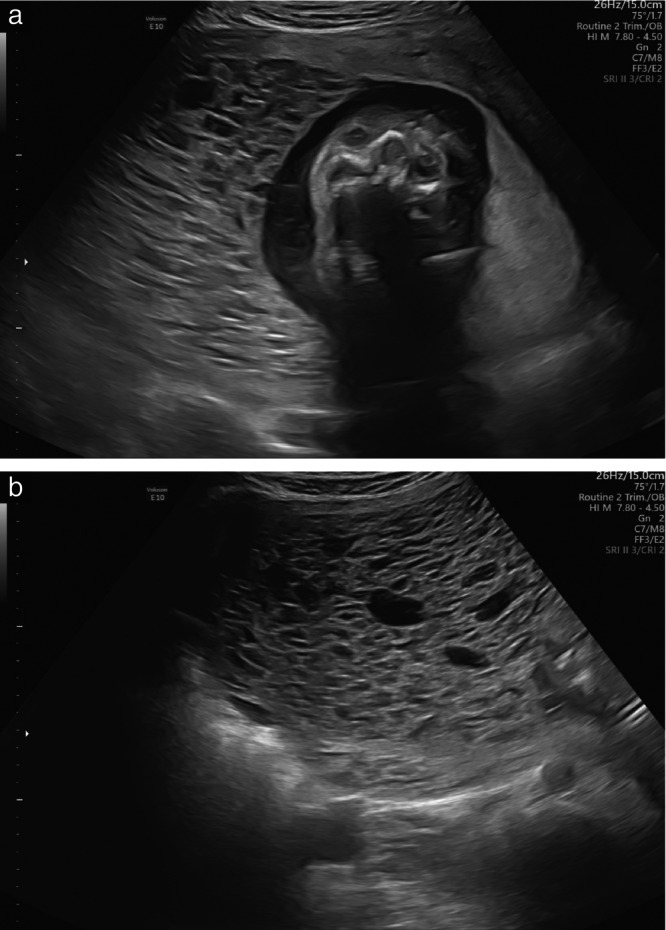
Grayscale transabdominal ultrasound images of original case of complete hydatidiform mole and coexisting normal fetus seen at our center at 18 weeks of gestation. (a) Molar placenta and normal placenta are visible to left and right, respectively, of fetal skull. (b) Magnification of enlarged molar placenta with microcystic appearance.

**Figure 5:**
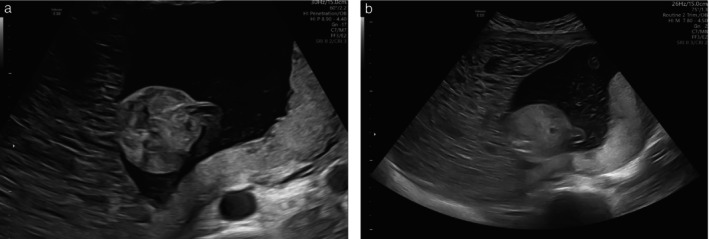
Grayscale transabdominal ultrasound images at different levels of magnification from original case of complete hydatidiform mole and coexisting normal fetus seen at our center at 18 weeks of gestation. Molar placenta appears as bulky, multicystic mass on left side of images; normal placenta and fetal abdomen are also visible. (a) Higher magnification shows detailed ultrasound features of both molar and normal placenta. (b) Lower magnification illustrates complete section of the two placentas.

**Figure 6:**
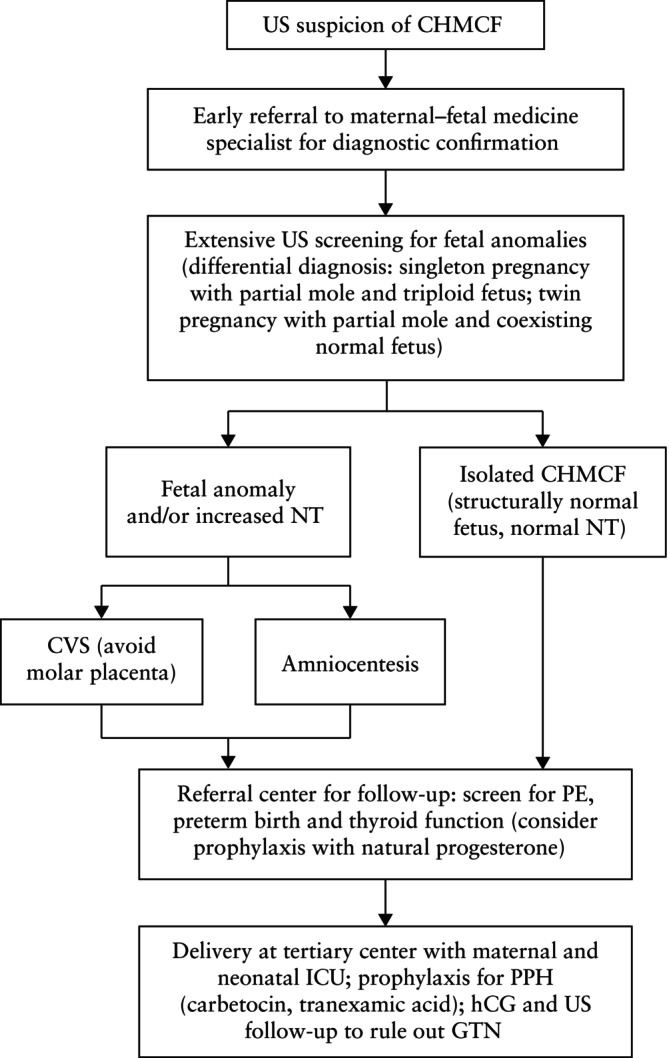
Algorithm for prenatal management of complete hydatidiform mole and coexisting normal fetus (CHMCF). CVS, chorionic villus sampling; GTN, gestational trophoblastic neoplasia; hCG, human chorionic gonadotropin; ICU, intensive care unit; NT, nuchal translucency; PE, pre‐eclampsia; PPH, postpartum hemorrhage; US, ultrasound.

This meta‐analysis presents the obstetric and oncological outcomes of 417 pregnancies with CHMCF pooled from 19 studies. A little under half of the cases underwent TOP, while slightly over half elected to continue their pregnancy, with no conclusive benefit of TOP on the risk of subsequent GTN. The risk of miscarriage was about 33% and the risk of preterm birth < 37 weeks was around 68%, most of which were late preterm births (only 12% of births occurred before 32 weeks). Macroscopic and ultrasound findings in original cases seen at the IRCCS San Raffaele Scientific Institute, Milan, Italy, are shown in Figures 3, 4, 5. The suggested algorithm for prenatal management of CHMCF cases based on our results is shown in Figure 6.

### Interpretation

The timing of diagnosis of CHMCF varied, with 53% of cases diagnosed in the first trimester. This variability reflects the wide timespan in publication date of the included studies and improvement in diagnostics over time, yet early diagnosis of CHMCF remains challenging due to a lack of distinct sonographic features. Although most cases in this meta‐analysis were diagnosed via ultrasound, a case series from our group reported that, in all cases of CHMCF that underwent first‐trimester ultrasound examination, placental abnormalities were initially misdiagnosed as subchorionic hematoma^22^. This highlights the need for early ultrasound evaluation by experienced specialists.

The pooled prevalence of elective TOP was 49%, with only 6% attributable to maternal complications. The rate of maternal request for TOP varies according to national abortion laws, cultural and religious differences, and local counseling practice. While TOP was considered historically to be the only management option for CHMCF^38^, reports of live births emerged in the early 2000s^36^. Current management approaches promote individualized assessment based on symptoms^39^. TOP becomes necessary in the case of maternal complications such as recurrent bleeding or early‐onset pre‐eclampsia, which occur in up to 20% of CHMCF cases^40^ compared with 0.2% in the general population^41^. Notably, one study included in this meta‐analysis reported a maternal death due to severe acute respiratory insufficiency during a medically induced TOP for severe pre‐eclampsia, which the authors attributed to massive trophoblastic embolization and pulmonary edema^30^.

CHMCF pregnancies have a higher prevalence of SGA (41% in our synthesis) compared with the general population (7–10%)^42^. Although CHMCF pregnancies are dichorionic, the molar mass may disrupt placentation and vascular supply to the normal fetus, causing fetal growth restriction and consequent stillbirth in undetected or poorly managed cases. An imbalance between pro‐ and antiangiogenic factors has also been described^43^.

Preterm birth is a major determinant of neonatal morbidity and mortality. Neonatal and maternal death were reported rarely by the studies included in our meta‐analysis. One study reported eight neonatal deaths, primarily due to prematurity^1^. In our meta‐analysis, lack of data from the original studies precluded calculation of pooled estimates based on the etiology of preterm birth, i.e. spontaneous *vs* iatrogenic due to maternal health deterioration. This knowledge gap should be addressed by future studies because the lack of understanding of the pathophysiology of preterm birth impedes the development of strategies for management or prevention^44^. Referral of women with CHMCF to a tertiary facility may prove decisive for pregnancy management and neonatal care in the case of threatened preterm birth.

The clinical presentation of hydatidiform mole has evolved with advances in ultrasound. The typical features of increased uterine volume, ovarian theca lutein cysts and vaginal bleeding, once common in hydatidiform mole, are now less frequent due to early diagnosis^45^. In CHMCF, clinical characteristics resemble those of late hydatidiform mole and the ovaries may be enlarged due to hyperreactio luteinalis^7^, ^46^. CHM pregnancies are associated with a 13–20% risk of GTN^47^, ^48^. The reported rate of GTN following CHMCF varies: Niemann *et al*.^35^ found a 25% incidence, which was not significantly higher than that following a singleton CHM pregnancy (17%) and Steller *et al*.^10^ found a rate of 63%, compared with only 10% of CHM patients. Hajri *et al*.^1^, the largest series in this review, reported an incidence of GTN of 26%, whereas Lin *et al*.^30^ and Lu *et al*.^31^ reported incidences as high as 46%. These discrepancies result partly from whether cases were diagnosed before or after the introduction of the FIGO criteria in 2002^9^, as well as from differences in ethnicity and potential selection bias.

The timing of TOP may also affect the risk of developing GTN. Sebire *et al*.^36^ reported similar rates of GTN between women who underwent first‐trimester TOP (16% (95% CI, 3–39%)) and those who progressed into the second/third trimester (21% (95% CI, 11–33%)). In the present meta‐analysis, 34% of CHMCF cases developed GTN during follow‐up, with a small difference in risk between continued pregnancy (20%) and elective TOP (14%). Uncomplicated cases appeared to have a reduced risk of GTN (6%).

The association between GTN, need for medical TOP due to severe complications and higher hCG levels could be attributed to aggressive behavior of trophoblastic tissue causing maternal morbidity. In contrast, an almost silent disease results in an uncomplicated pregnancy. There have been previous attempts to link outcomes to hCG concentrations. While identifying a cut‐off value for maximum serum hCG or hCG at diagnosis proved inconclusive, longitudinal trends in hCG were shown to differ between uncomplicated and unsuccessful pregnancies, decreasing in the former and increasing in the latter^26^. This observation could serve as a predictive factor in the management of CHMCF, without the need for universal reference intervals for hCG levels.

Reassuringly, the majority of GTN cases following CHMCF in this analysis were low risk and were treated successfully with single‐agent chemotherapy.

### Strengths and limitations

To date, this is the largest systematic review and meta‐analysis to summarize clinical outcomes in pregnancies with CHMCF. It provides robust pooled estimates of outcomes with approximately double the sample size of the most recent meta‐analysis on this subject^49^. By including only cases with histopathological confirmation of CHMCF diagnosis, we excluded the risk of bias deriving from inclusion of partial molar disease or live fetuses with other types of placental anomaly. This analysis should provide valuable support to current clinical practice and has identified etiology of preterm birth in CHMCF as a priority for future research.

The risk of bias in this meta‐analysis arises from the retrospective design of the cohort studies (seven studies, 325 cases) and the inclusion of case series with small sample sizes (12 studies, 92 cases), although 10/12 studies reported complete and consecutive case inclusion during their respective study periods. It should be noted that the study of Hajri *et al*.^1^, being the largest and most recent, may have had a substantial impact on the pooled estimates. Nevertheless, sensitivity analyses including only cohort studies yielded consistent results. Moreover, most of the case series are hospital‐based, rather than national cohorts, so are subject to case ascertainment bias. Additionally, the significant variability in publication date, CHMCF management and definition of GTN between studies make comparisons challenging. Furthermore, none of the studies provided information on the counseling offered to couples. Finally, both statistical and clinical heterogeneity may be significantly influenced by the wide range of gestational ages at diagnosis.

### Conclusion

CHMCF pregnancies present a high risk of maternal, obstetric and neonatal complications, including miscarriage, pre‐eclampsia, SGA and postpartum hemorrhage. A strong association with preterm birth < 37 and < 32 weeks was found, albeit with a lack of data on the etiology of preterm birth. The risk of GTN is not clearly mitigated by TOP; the slight difference in prevalence of GTN between groups does not provide definitive evidence of benefit and should be interpreted with caution given the heterogeneity in outcome definitions and study protocols. TOP should therefore be discussed with patients but not recommended for improving maternal outcome. In any case, the final decision to terminate or continue a pregnancy with CHMCF is likely influenced by factors beyond clinical indications, including cultural, social, economic, religious and personal factors. An international registry of CHMCF may be proposed to advance research. Early referral to a maternal–fetal medicine unit with expertise in trophoblastic disease is essential for pregnancy management by a multidisciplinary team. Screening protocols for preterm birth and pre‐eclampsia should be implemented to enable timely intervention. Postnatal maternal follow‐up should include hCG and ultrasound monitoring to detect early signs of GTN development^50^.

## Supporting information


**Appendix S1** Search strategy.

**Table S1** Quality assessment of cohort studies using the Newcastle–Ottawa scale.
**Table S2** Quality assessment of case series using the Joanna Briggs Institute checklist.
**Table S3** Sensitivity analysis of pooled proportions of clinical outcomes in complete hydatidiform mole and coexisting normal fetus, restricted to cohort studies (*n* = 325 pregnancies).
**Table S4** GRADE assessment of included studies.

**Figure S1** Forest plots of pooled proportions of obstetric and oncological outcomes in complete hydatidiform mole and coexisting normal fetus.

## Data Availability

Data sharing is not applicable to this article as no new data were created or analyzed in this study.
