# Supplementary material for: Multiple pregnancy with complete hydatidiform mole and coexisting normal fetus: systematic review and meta‐analysis of clinical outcomes from non‐randomized studies
Source: Ultrasound Obstet Gynecol. 2025 Oct 9;67(3):272–82. doi: 10.1002/uog.70104 (PMC12951261; doi:10.1002/uog.70104)
Supplement: Supplementary file 1 — Appendix S1 Search strategy. [file UOG-67-272-s001.docx]

**Appendix S1.** Search strategy

1. **PubMed**

((molar pregnancy AND viable fetus) OR (hydatidiform mole AND coexisting fetus) OR (Hydatiform mole AND coexisting fetus))

complete mole

((obstetric outcome) OR (complications) OR (management) OR (perinatal outcome))

| **Search** | **Query** | **Items found** |
| --- | --- | --- |
| **#7** | #4 AND #3 | 240 |
| **#4** | #1 AND #2 | 334 |
| **#3** | ((obstetric outcome) OR (complications) OR (management) OR (perinatal outcome)) | 7,447,344 |
| **#2** | complete mole | 6,865 |
| **#1** | ((molar pregnancy AND viable fetus) OR (hydatidiform mole AND coexisting fetus) OR (Hydatiform mole AND coexisting fetus)) | 341 |

1. **Embase**

'molar pregnancy':ti,ab,kw AND 'viable fetus':ti,ab,kw OR ('hydatidiform mole':ti,ab,kw AND 'coexisting fetus':ti,ab,kw) OR ('hydatiform mole':ti,ab,kw AND 'coexisting fetus':ti,ab,kw)

'complete mole':ti,ab,kw

'obstetric outcome':ti,ab,kw OR complications:ti,ab,kw OR management:ti,ab,kw OR 'perinatal outcome':ti,ab,kw

| **Search** | **Query** | **Items found** |
| --- | --- | --- |
| **#7** | #4 AND #3 | 25 |
| **#4** | #1 AND #2 | 36 |
| **#3** | 'obstetric outcome':ti,ab,kw OR complications:ti,ab,kw OR management:ti,ab,kw OR 'perinatal outcome':ti,ab,kw | 3,462,673 |
| **#2** | 'complete mole':ti,ab,kw | 573 |
| **#1** | 'molar pregnancy':ti,ab,kw AND 'viable fetus':ti,ab,kw OR ('hydatidiform mole':ti,ab,kw AND 'coexisting fetus':ti,ab,kw) OR ('hydatiform mole':ti,ab,kw AND 'coexisting fetus':ti,ab,kw) | 125 |

1. **Scopus**

TITLE-ABS-KEY((molar pregnancy AND viable fetus) OR (hydatidiform mole AND coexisting fetus) OR (hydatiform mole AND coexisting fetus))

TITLE-ABS-KEY (Complete AND mole)

TITLE-ABS-KEY ((obstetric outcome) OR (complications) OR (management) OR (perinatal outcome))

| **Search** | **Query** | **Items found** |
| --- | --- | --- |
| **#7** | #4 AND #3 | 114 |
| **#4** | #1 AND #2 | 176 |
| **#3** | TITLE-ABS-KEY ((obstetric outcome) OR (complications) OR (management) OR (perinatal outcome)) | 7,963,289 |
| **#2** | TITLE-ABS-KEY (Complete AND mole) | 5,155 |
| **#1** | TITLE-ABS-KEY((molar pregnancy AND viable fetus) OR (hydatidiform mole AND coexisting fetus) OR (hydatiform mole AND coexisting fetus)) | 260 |
