# Supplementary material for: Multiple pregnancy with complete hydatidiform mole and coexisting normal fetus: systematic review and meta‐analysis of clinical outcomes from non‐randomized studies
Source: Ultrasound Obstet Gynecol. 2025 Oct 9;67(3):272–82. doi: 10.1002/uog.70104 (PMC12951261; doi:10.1002/uog.70104)
Supplement: Supplementary file 2 — Table S1 Quality assessment of cohort studies using the Newcastle–Ottawa scale. Table S2 Quality assessment of case series using the Joanna Briggs Institute checklist. Table S3 Sensitivity analysis of pooled proportions of clinical outcomes in complete hydatidiform mole and coexisting normal fetus, restricted to cohort studies (n = 325 pregnancies). Table S4 GRADE assessment of included studies. [file UOG-67-272-s003.docx]

**Table S1** Quality assessment of cohort studies using Newcastle–Ottawa scale

| **Study** | ***Selection*** | | | | ***Comparability*** | ***Outcome*** | | | ***NOS score*** |
| --- | --- | --- | --- | --- | --- | --- | --- | --- | --- |
|  | **Q1** | **Q2** | **Q3** | **Q4** | **Q5** | **Q6** | **Q7** | **Q8** |  |
| Fishman et al., 1998 | 1 | NA | 1 | 1 | NA | 1 | 1 | 1 | 6 |
| Hajri et al., 2024 | 1 | NA | 1 | 1 | NA | 1 | 1 | 1 | 6 |
| Hancock et al., 2006 | 1 | NA | 1 | 1 | NA | 1 | 1 | 1 | 6 |
| Liang et al., 2022 | 1 | 1 | 1 | 1 | 2 | 1 | 1 | 1 | 9 |
| Lin et al., 2017 | 1 | 1 | 1 | 1 | 2 | 1 | 1 | 1 | 9 |
| Niemann et al., 2007 | 1 | 1 | 1 | 1 | 2 | 1 | 1 | 1 | 9 |
| Sebire et al., 2002 | 1 | NA | 1 | 1 | NA | 1 | 1 | 1 | 6 |

Note: Q1: Representativeness of the exposed cohort; Q2: Selection of the nonexposed cohort; Q3: Ascertainment of exposure; Q4: Illustration that there was no result of interest at start of research; Q5: Comparability of cohort based on the design or analysis; Q6: Evaluation of result; Q7: Was follow up long enough for outcome to happen; Q8: Adequate of follow up of cohort.

**Table S2** Quality assessment of case series using Joanna Briggs Institute checklist

| **Study** | **Q1** | **Q2** | **Q3** | **Q4** | **Q5** | **Q6** | **Q7** | **Q8** | **Q9** | **Q10** |
| --- | --- | --- | --- | --- | --- | --- | --- | --- | --- | --- |
| Lu et al., 2022 | yes | yes | yes | yes | yes | yes | unclear | yes | unclear | yes |
| Marcorelles et al., 2005 | yes | yes | yes | yes | yes | yes | yes | yes | unclear | yes |
| McNally et al., 2021 | yes | yes | yes | yes | yes | yes | yes | yes | unclear | yes |
| Miller et al., 1993 | yes | yes | yes | unclear | unclear | yes | yes | yes | yes | yes |
| Zilberman et al., 2019 | yes | yes | yes | yes | yes | yes | yes | yes | yes | yes |
| Lee et al., 2010 | yes | yes | yes | yes | yes | yes | yes | yes | unclear | yes |
| Giorgione et al., 2017 | yes | yes | yes | yes | yes | yes | yes | yes | yes | yes |
| Kutuk et al., 2010 | yes | yes | yes | yes | yes | yes | yes | yes | yes | yes |
| Hemida et al., 2022 | yes | yes | yes | yes | yes | no | no | no | yes | yes |
| Steller et al., 1994 | yes | yes | yes | yes | yes | yes | yes | yes | yes | yes |
| Jauniaux et al., 1997 | yes | yes | yes | yes | yes | no | yes | no | yes | yes |
| Kihara et al., 2012 | yes | yes | yes | no | no | yes | yes | yes | yes | yes |

Notes: Q1: Were there clear criteria for inclusion in the case series?; Q2: Was the condition measured in a standard, reliable way for all participants included in the case series?; Q3: Were valid methods used for identification of the condition for all participants included in the case series?; Q4: Did the case series have consecutive inclusion of participants?; Q5: Did the case series have complete inclusion of participants?; Q6: Was there clear reporting of the demographics of the participants in the study?; Q7: Was there clear reporting of clinical information of the participants?; Q8: Were the outcomes or follow-up results of cases clearly reported?; Q9: Was there clear reporting of the presenting site(s)/clinic(s) demographic information?; Q10: Was statistical analysis appropriate?

**Table S3** Sensitivity analysis of pooled proportions of clinical outcomes in complete hydatidiform mole and coexisting normal fetus, restricted to cohort studies (n = 325 pregnancies)

| Obstetrical and oncological outcomes | Number of studies | Pooled prevalence (%, per 100 obs) | 95% confidence intervals (CIs) | Heterogeneity (I2) |
| --- | --- | --- | --- | --- |
| *Diagnosis: timing and symptoms* |  |  |  |  |
| Symptoms at diagnosis  Symptoms at diagnosis (any)  Vaginal bleeding | 3  3 | 81.0%  62.0% | 33.0-100.0%  31.0-89.0% | 83.8%  60.2% |
| Suspected diagnosis by US evaluation | 4 | 68.0% | 60.0-76.0% | 0.0% |
| 1^st^ trimester diagnosis | 3 | 67.0% | 59.0-74.0% | 0.0% |
| *Pregnancy: course and complications* |  |  |  |  |
| TOP  TOP (total)  TOP on maternal request  TOP for maternal complications | 7  7  7 | 41.0%  26.0%  7.0% | 20.0-63.0%  9.0-48.0%  0.5-18.0% | 90.4%  90.9%  79.8% |
| Continuation of pregnancy | 7 | 75.0% | 52.0-92.0% | 91.5% |
| Spontaneous termination of pregnancy*  Miscarriage (<24 weeks)  IUFD (>24 weeks) | 7  7 | 33.0%  0.01% | 26.0-40.0%  0.01–5.0% | 0.0%  53.3% |
| Maternal complications*  Hypertensive diseases (any)  Pre-eclampsia  Hyperthyroidism | 5  5  3 | 14.0%  11.0%  7.0% | 2.0-34.0%  1.0-29.0%  0.1-18.0% | 81.7%  80.1%  0.0% |
| *Delivery: gestational age, mode, and complications* |  |  |  |  |
| Live birth* | 7 | 47.0% | 38.0-57.0% | 22.7% |
| Gestational age at delivery†  Term birth (≥37 weeks)  Pre-term birth (<37 weeks)  Very pre-term birth (<32 weeks) | 4  4  4 | 25.0%  75.0%  9.0% | 8.0-46.0%  54.0-92.0%  0.1-46.0% | 0.0%  0.0%  30.9% |
| Mode of delivery/labour†  Caesarean section  Vaginal birth  Iatrogenic delivery (for maternal complications) | 4  4  4 | 88.0%  12.0%  9.0% | 33.0-100.0%  0.01-67.0%  0.01-46.0% | 0.0%  0.0%  30.9% |
| Neonatal complications†  Neonatal mortality | 5 | 0.01% | 0.01–9.0% | 50.6% |
| Maternal complications (peri-partum) †  Maternal mortality | 5 | 0.0% | 0.0-0.0% | 0.0% |
| *GTN (with subgroups by pregnancy management) ‡* |  |  |  |  |
| GTN (total)  GTN in elective TOP  GTN in continued pregnancies  GTN in pregnancies ending with a living baby | 7  5  5  4 | 33.0%  15.0%  19.0%  8.0% | 22.0-44.0%  4.0-30.0%  8.0-32.0%  2.0-15.0% | 61.1%  75.7%  65.9%  0.0% |

Abbreviations: US, ultrasound; TOP, termination of pregnancy; IUFD, intrauterine fetal death; SGA, small for gestational age. GTN, gestational trophoblastic neoplasia. Notes: ^*^Proportions calculated out of the number of those who continued their pregnancy. †Proportions calculated out of the total number of live births. ‡Proportions calculated out of the total number of patients with reported follow-up for diagnosis of GTN.

**Table S4** GRADE assessment of included studies

| **GRADE assessment** | | | | | **Summary of findings** |
| --- | --- | --- | --- | --- | --- |
| Limitations | Inconsistency | Indirectness | Imprecision | Publication bias |  |
| serious limitations (observational studies and case series; limited sample size) | no inconsistency (no heterogeneity): Hyperthyroidism, Caesarean section, Vaginal birth, Low birth weight (<10°), Maternal mortality  low inconsistency (low heterogeneity): Vaginal bleeding at diagnosis, Hypertensive disorders at diagnosis, Vaginal bleeding after continuation of pregnancy, Live birth, Term birth (>37 weeks), Pre-term birth (<37 weeks), Very pre-term birth (<32 weeks)  moderate inconsistency (moderate heterogeneity): miscarriage (<24 weeks), IUFD (>24 weeks), Neonatal mortality  high inconsistency (moderate to high heterogeneity): all other investigated outcomes | moderate indirectness (use of substitute/surrogate endpoints, not accurate definition of outcomes, limited data on follow-up after pregnancy): all oncological outcomes, specifically: GTN (total), GTN in elective TOP, GTN in continued pregnancies, GTN in pregnancies ending with a living baby  no serious indirectness: all obstetrical outcomes investigated | high imprecision (lower to upper CIs >30%): Hyperemesis gravidarum at diagnosis, Suspected diagnosis by US evaluation, 1^st^ trimester diagnosis, TOP (total), TOP on maternal request, Continuation of pregnancy, Hyperemesis gravidarum after continuation of pregnancy, Term birth (>37 weeks), Pre-term birth (<37 weeks), Very pre-term birth (<32 weeks), Low birth weight (<10°), Caesarean section, Vaginal birth, Iatrogenic labour (for maternal complications), Post-partum haemorrhage  no serious imprecision (lower to upper CIs <30%): all other investigated outcomes | - | Low to Very low |

Abbreviations: IUFD, intrauterine fetal death; CIs, confidence intervals; US, ultrasound.
