# Supplementary material for: Multiple pregnancy with complete hydatidiform mole and coexisting normal fetus: systematic review and meta‐analysis of clinical outcomes from non‐randomized studies
Source: Ultrasound Obstet Gynecol. 2025 Oct 9;67(3):272–82. doi: 10.1002/uog.70104 (PMC12951261; doi:10.1002/uog.70104)

**Figure S1.** Forest plots of pooled proportions of obstetric and oncological outcomes in complete hydatidiform mole and coexisting normal fetus

1. Symptoms at diagnosis (any)


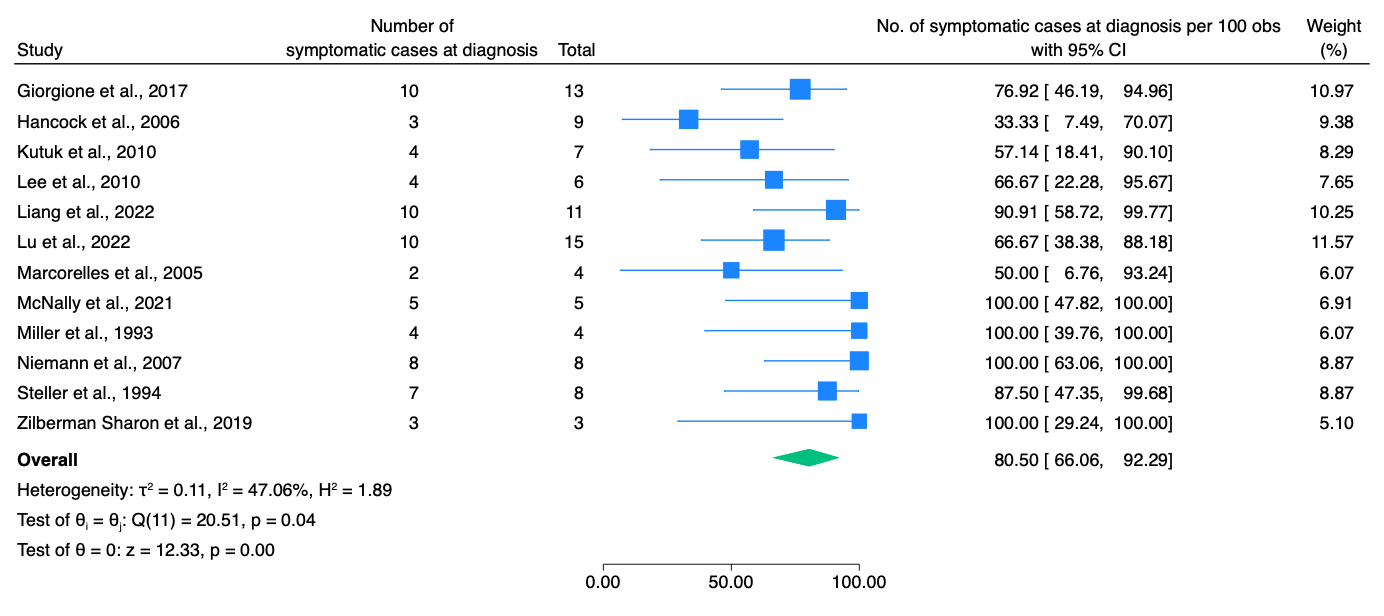


1. Vaginal bleeding


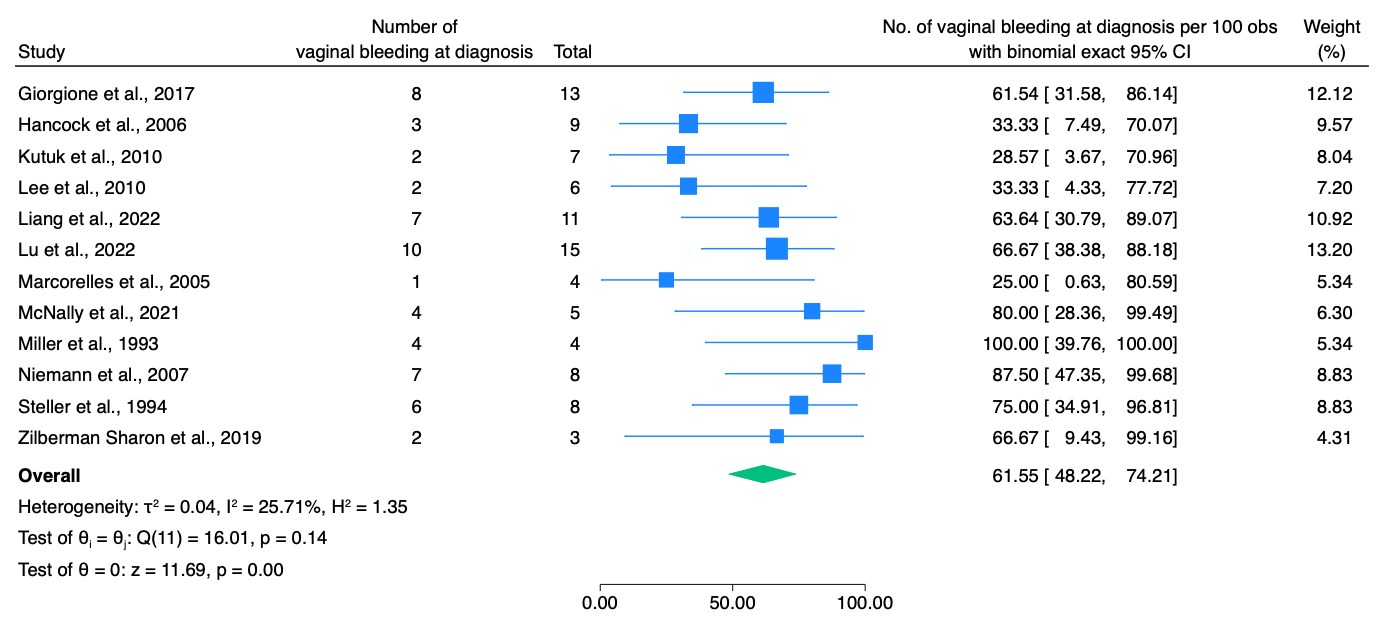


1. Hyperemesis gravidarum


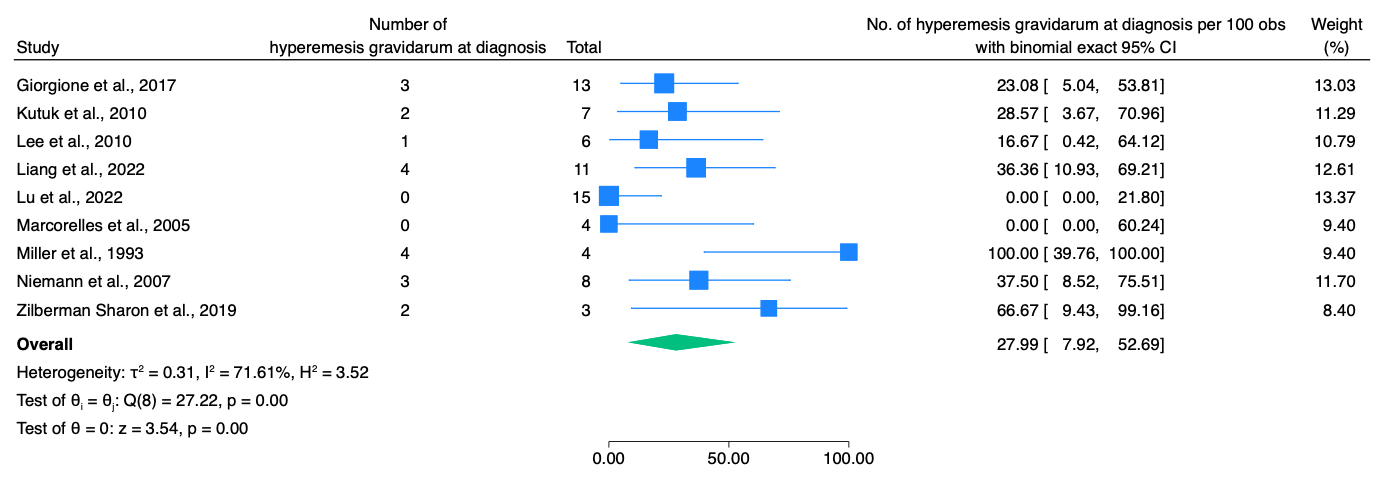


1. Hypertensive disorders


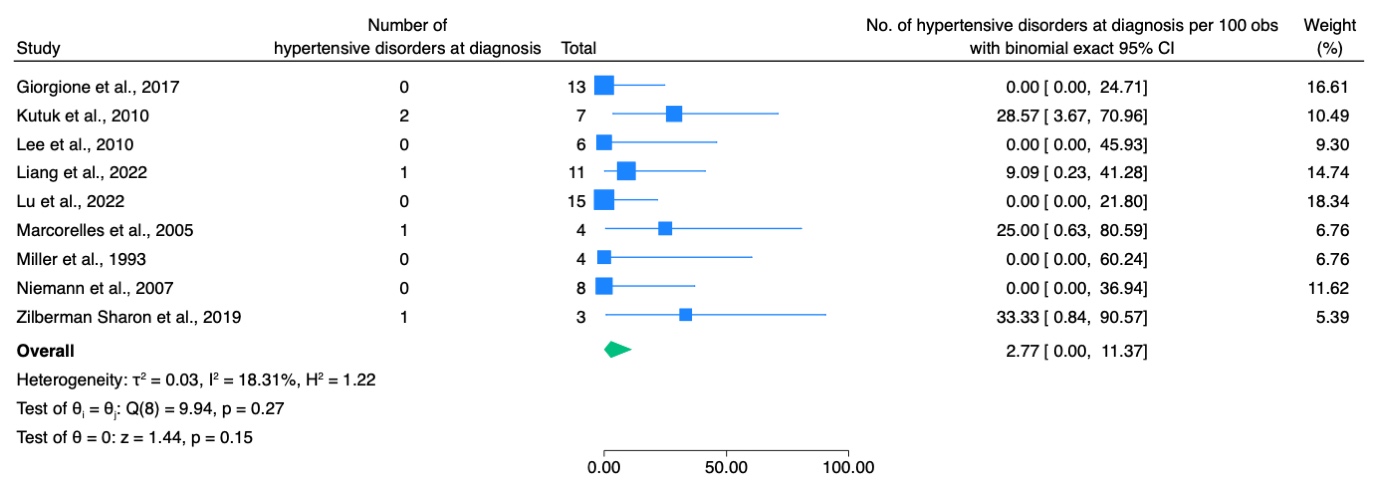


1. Suspected diagnosis by US evaluation


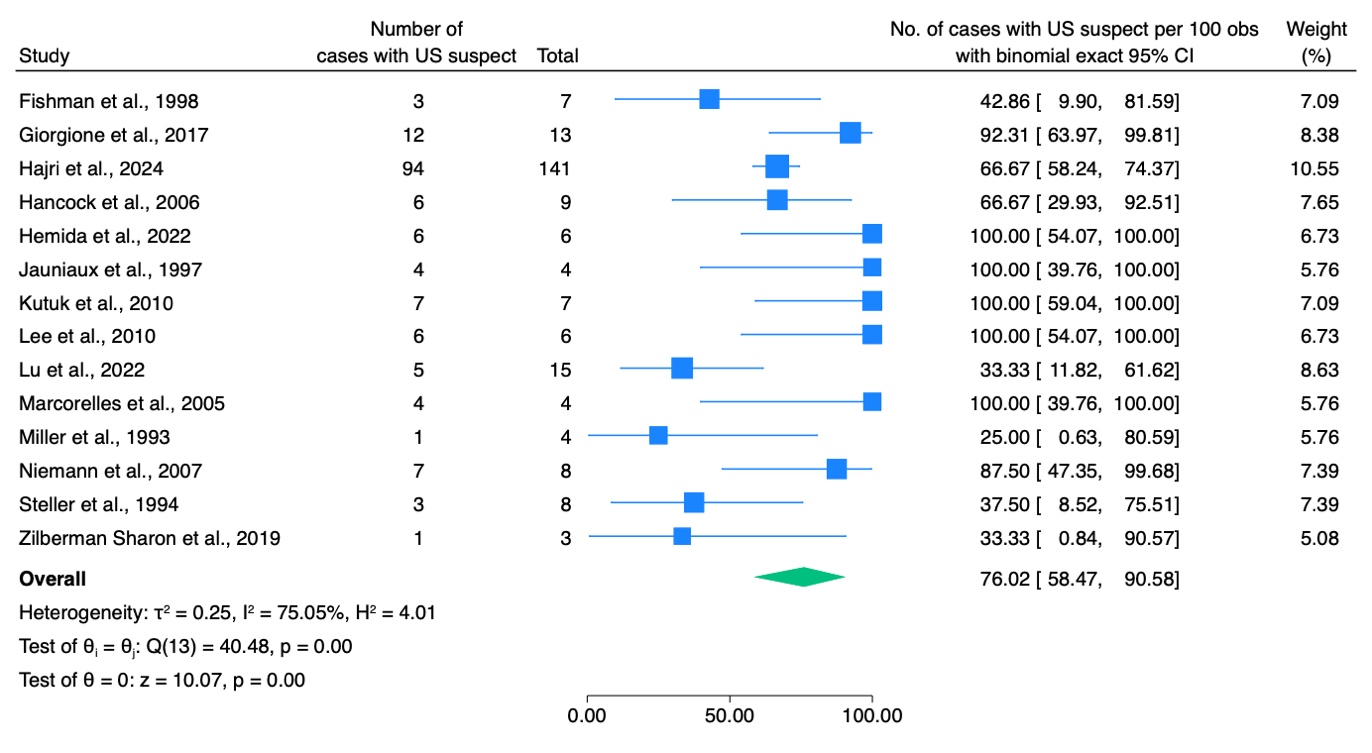


1. 1^st^ trimester diagnosis


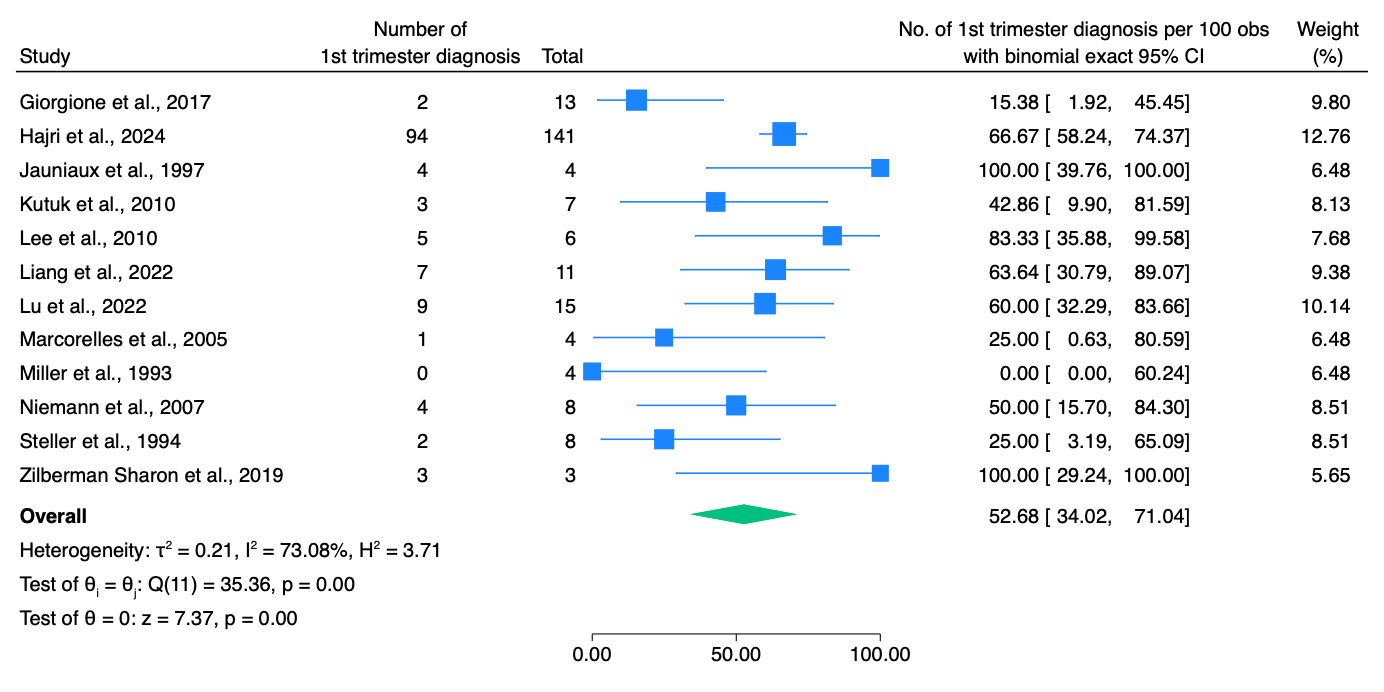


1. TOP (any)


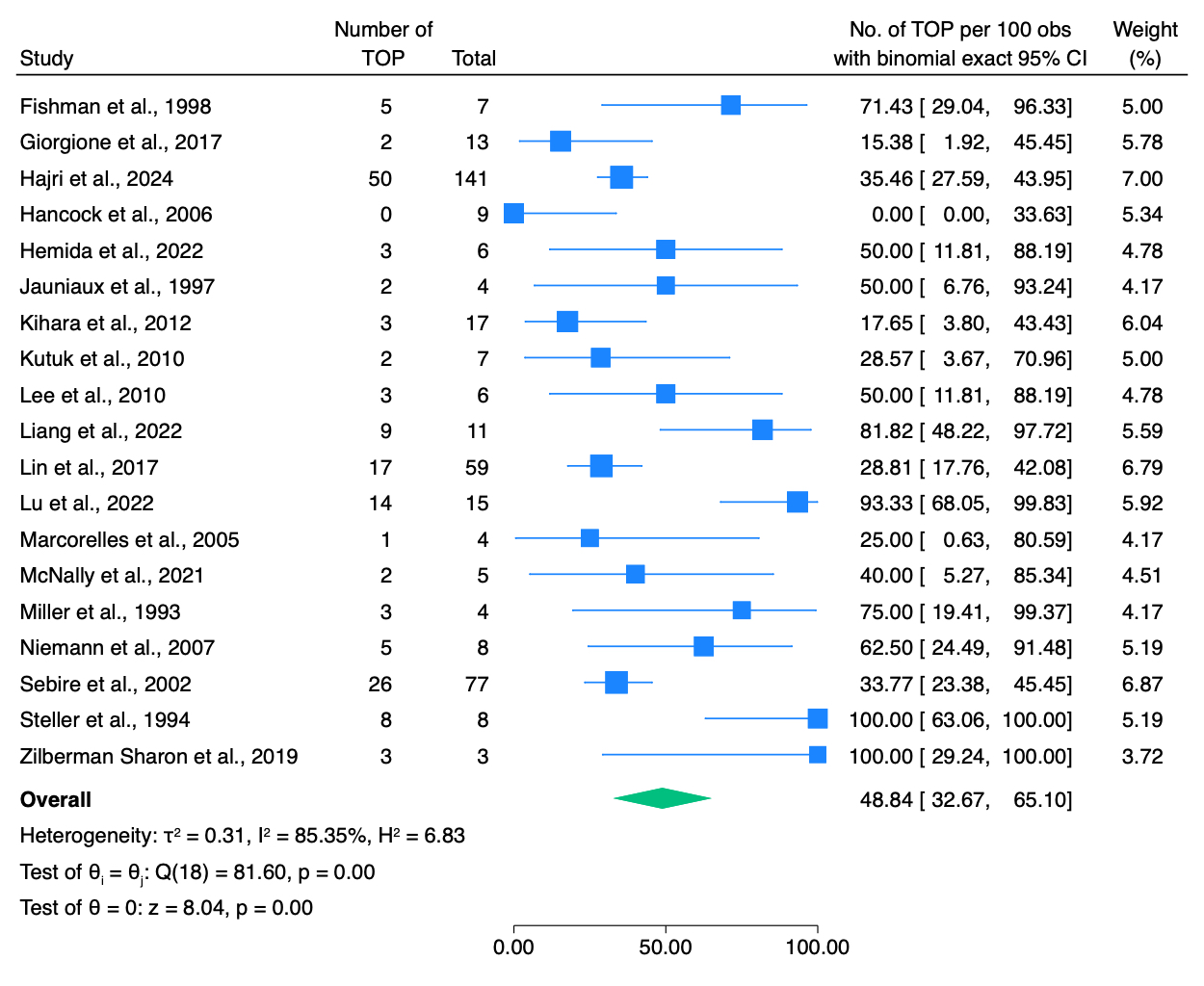


1. TOP on maternal request


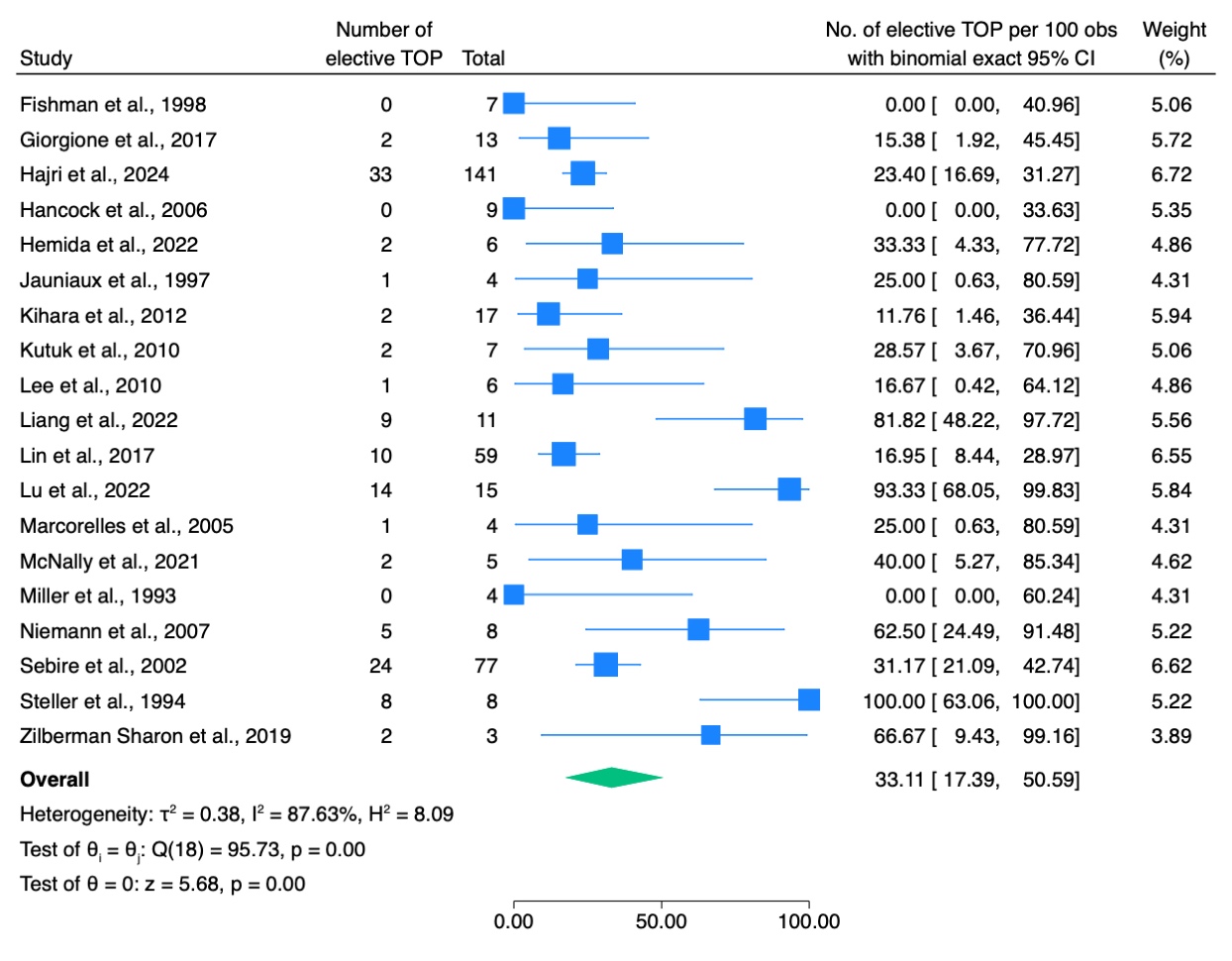


1. TOP for maternal complications


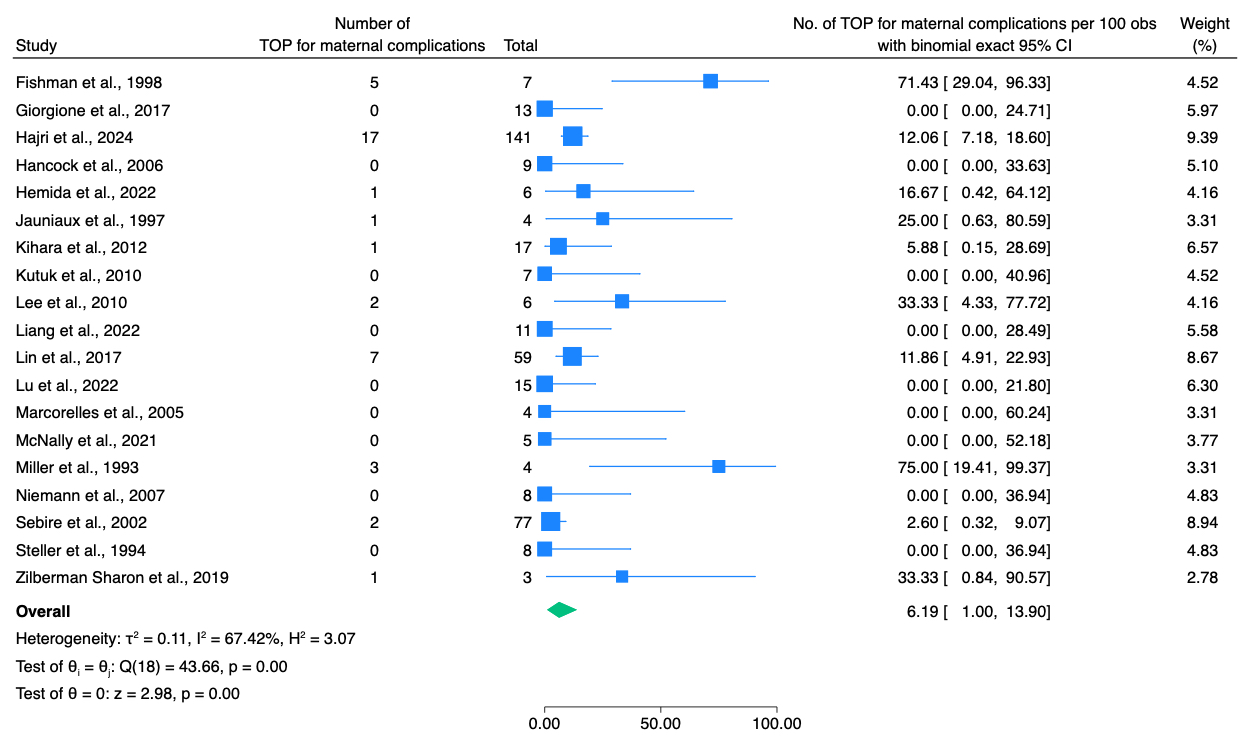


1. Continuation of pregnancy


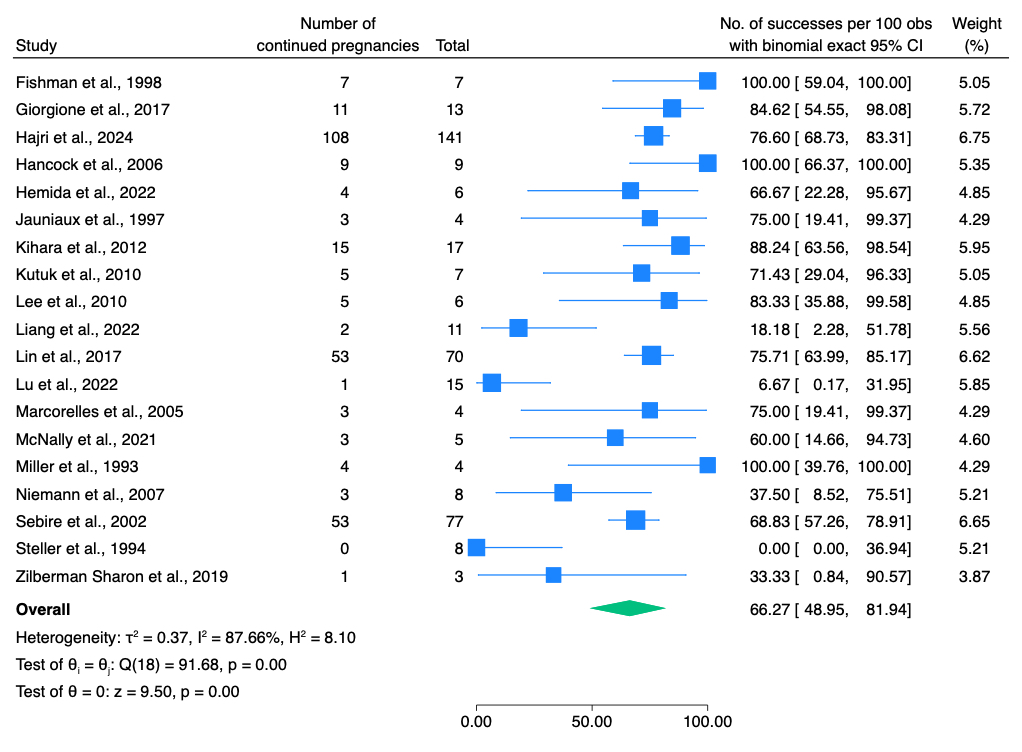


1. Miscarriage (<24 weeks)


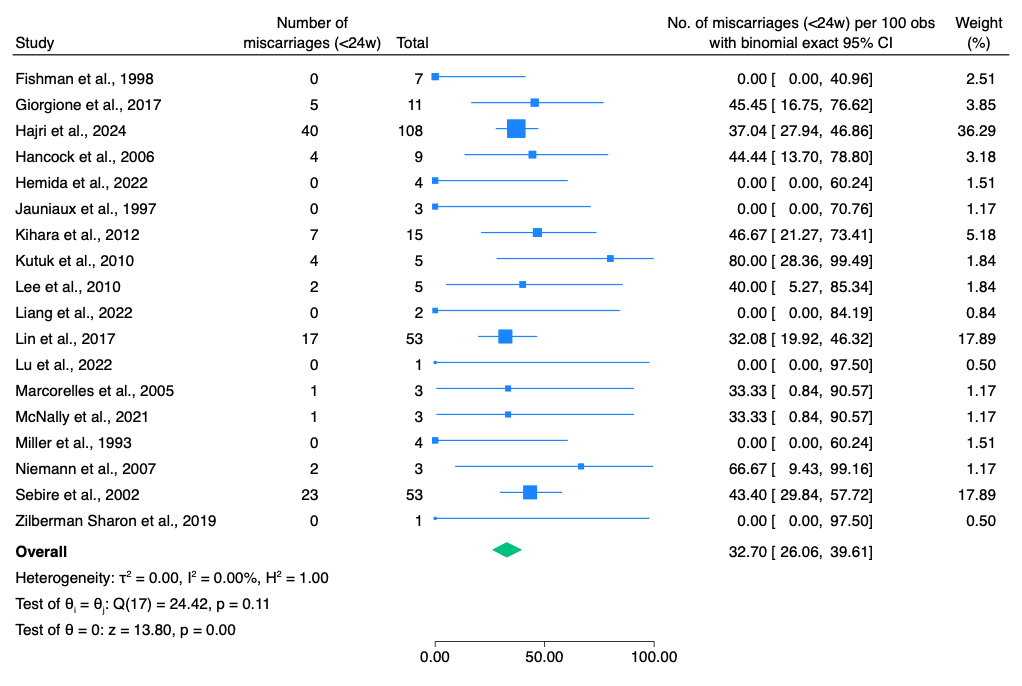


1. IUFD (≥24 weeks)


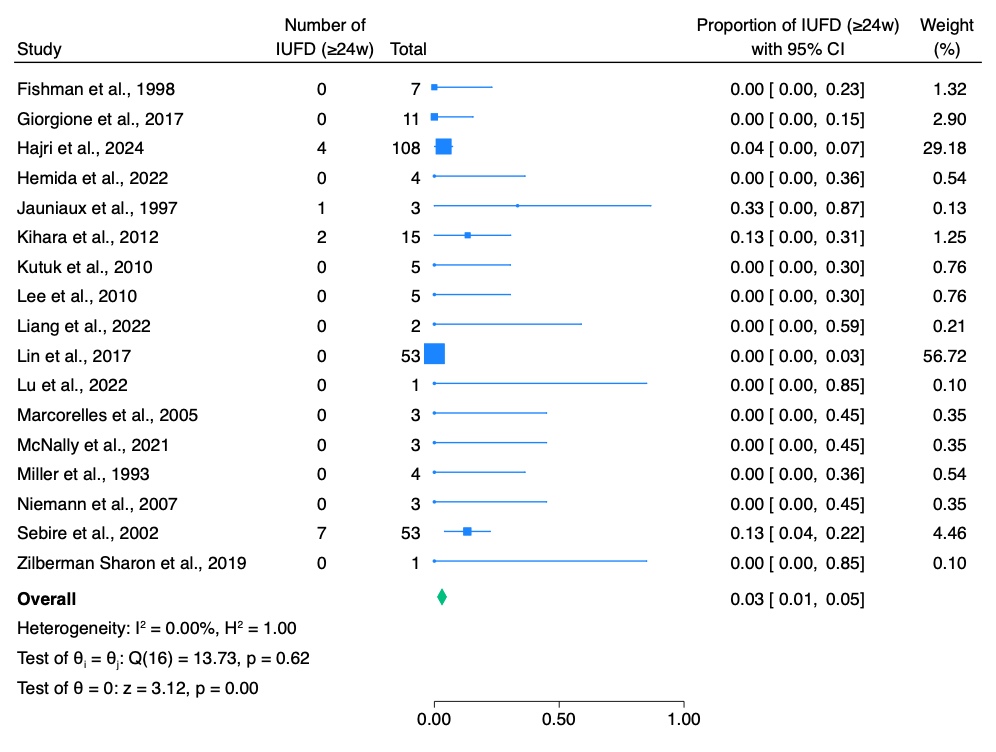


1. maternal complications: hypertensive diseases


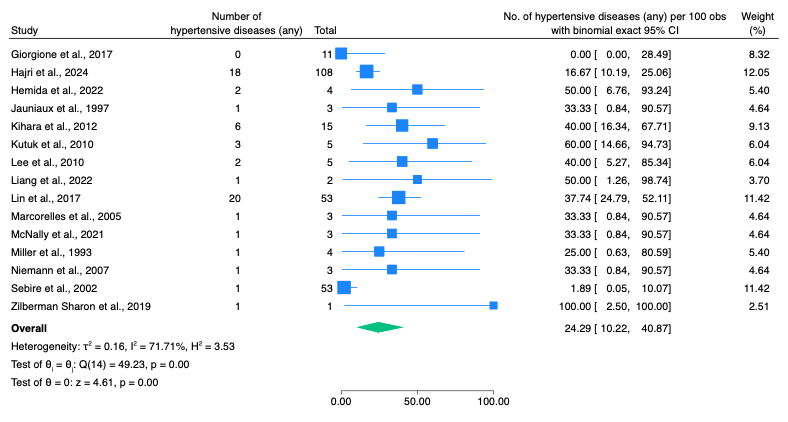


1. maternal complications: pre-eclampsia


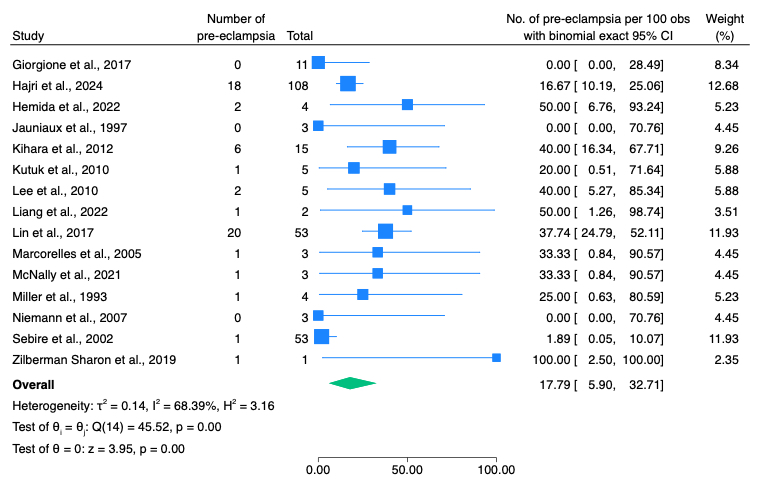


1. maternal complications: hyperthyroidism


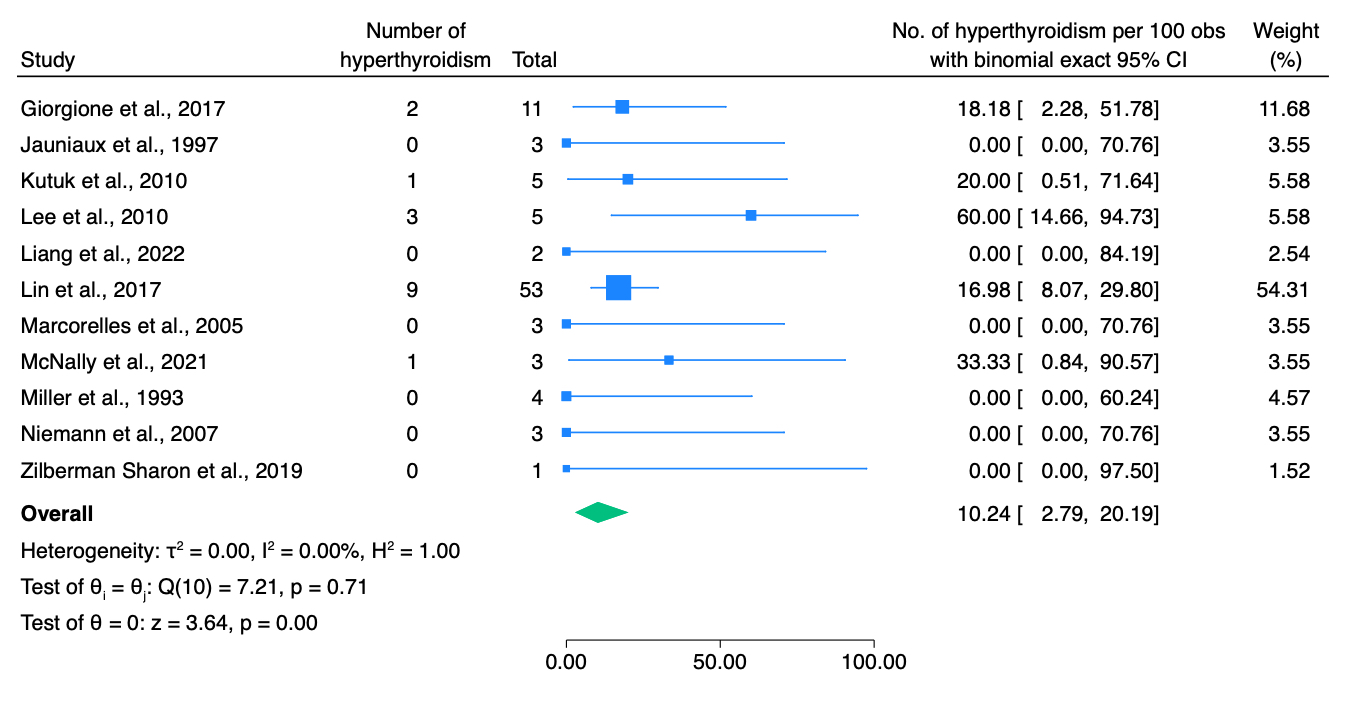


1. maternal complications: hyperemesis gravidarum


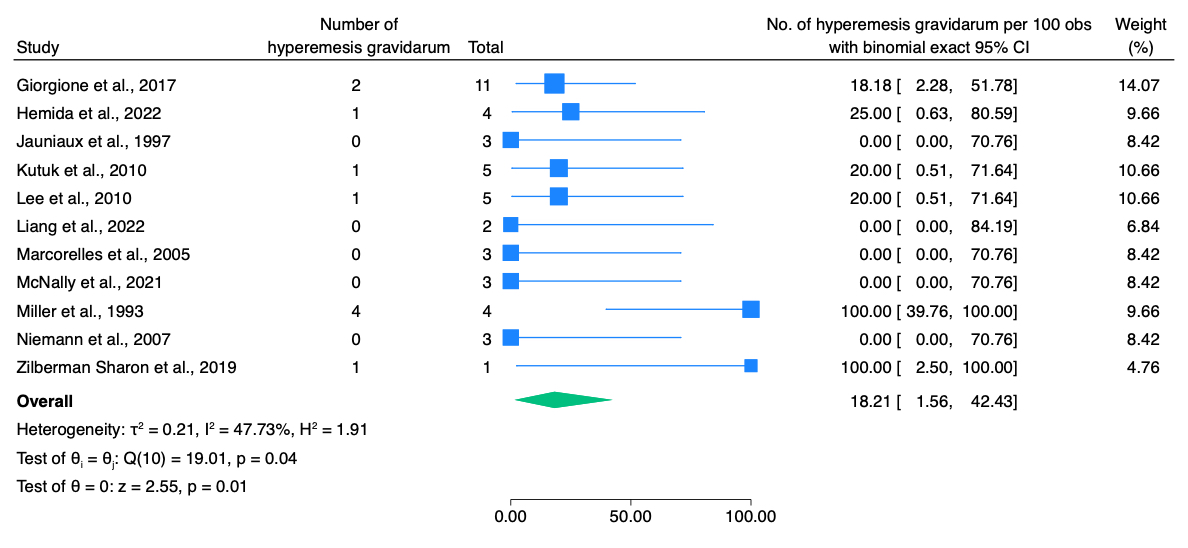


1. maternal complications: vaginal bleeding


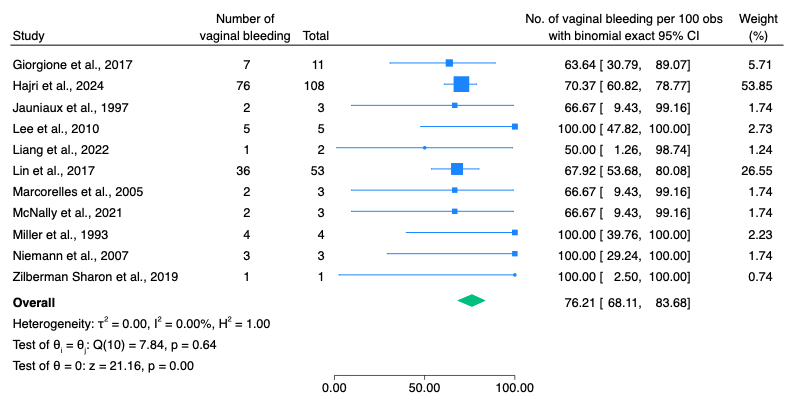


1. live birth


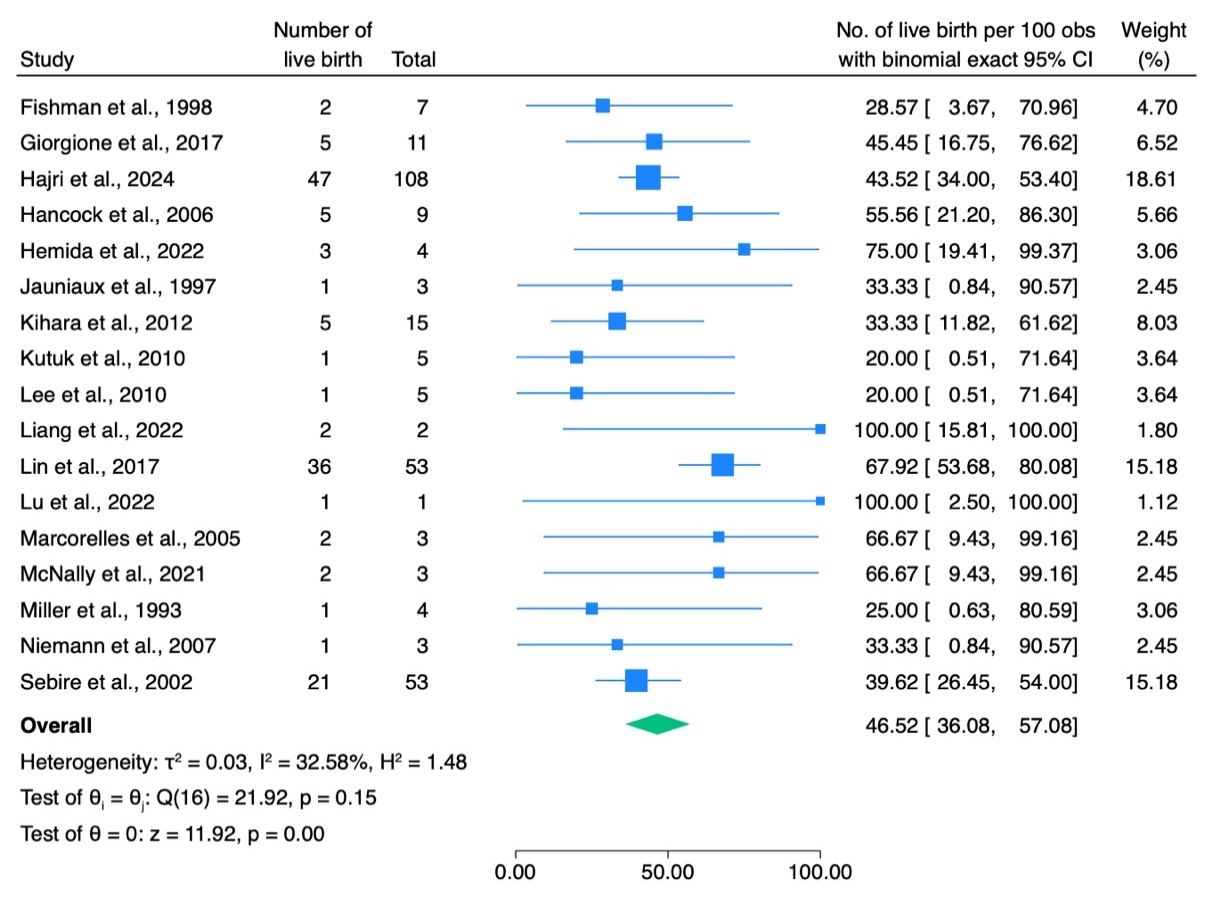


1. gestational age at delivery: term birth (≥37 weeks)


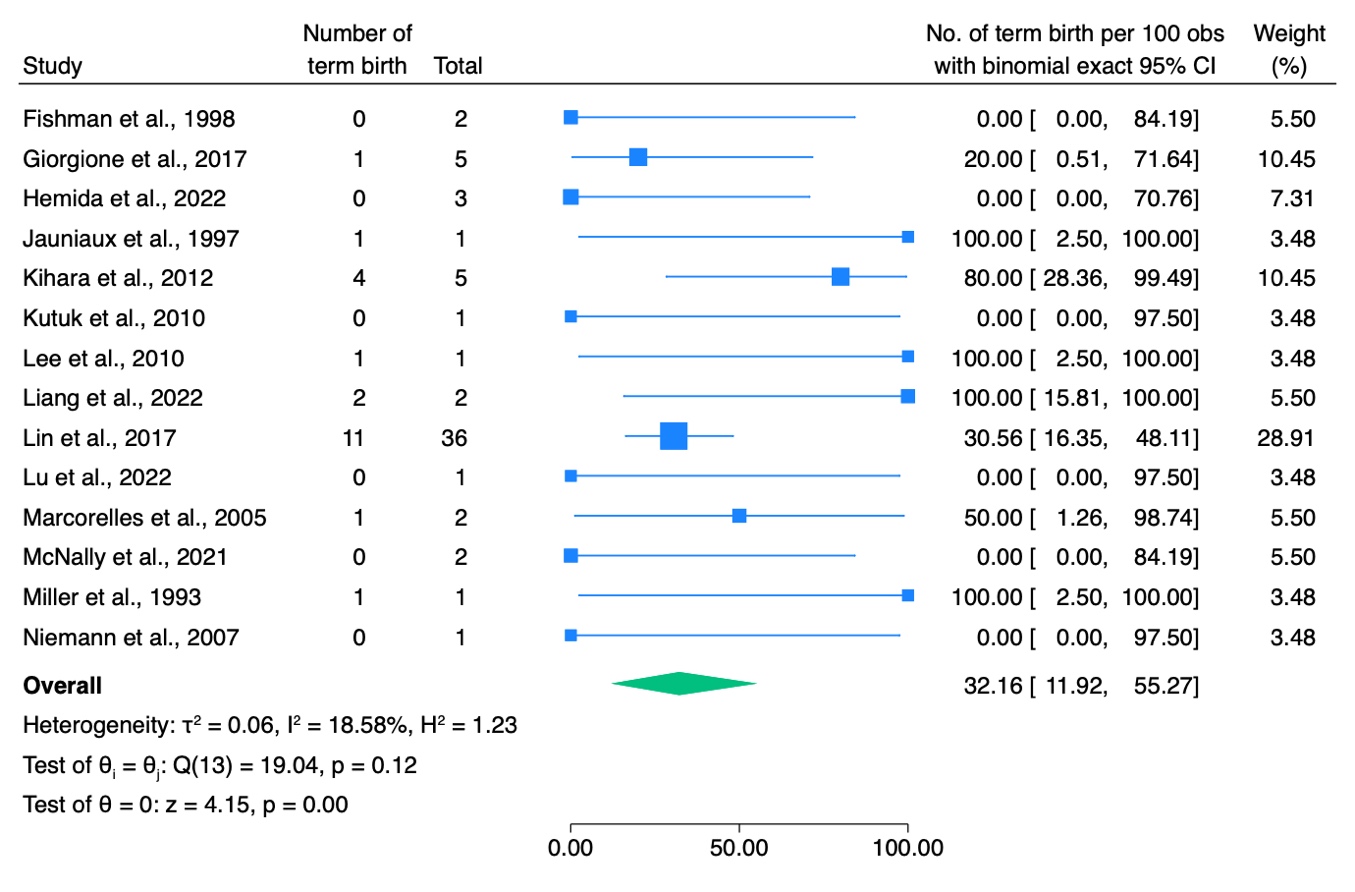


1. gestational age at delivery: pre-term birth (<37 weeks)


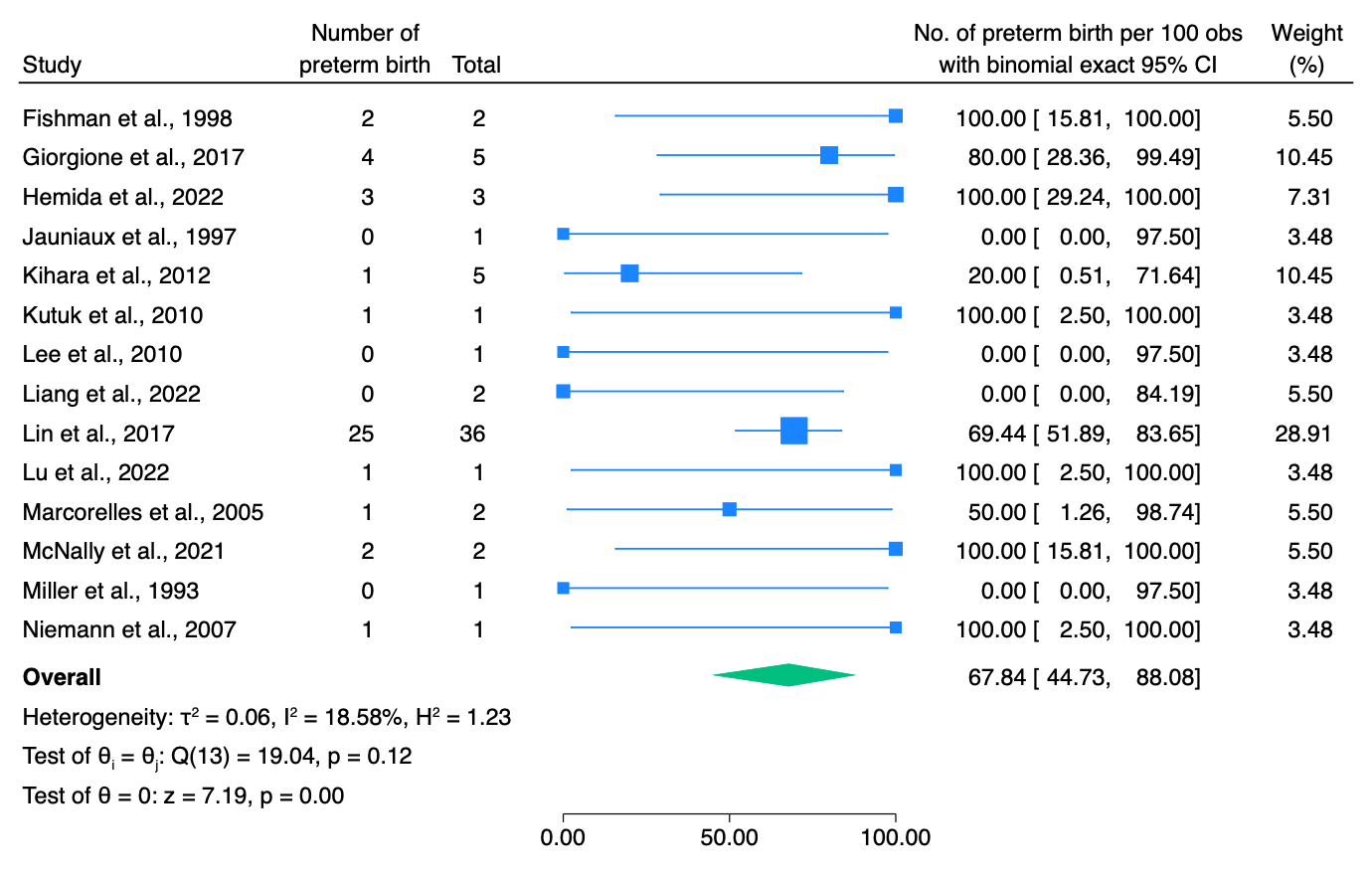


1. gestational age at delivery: very pre-term birth (<32 weeks)


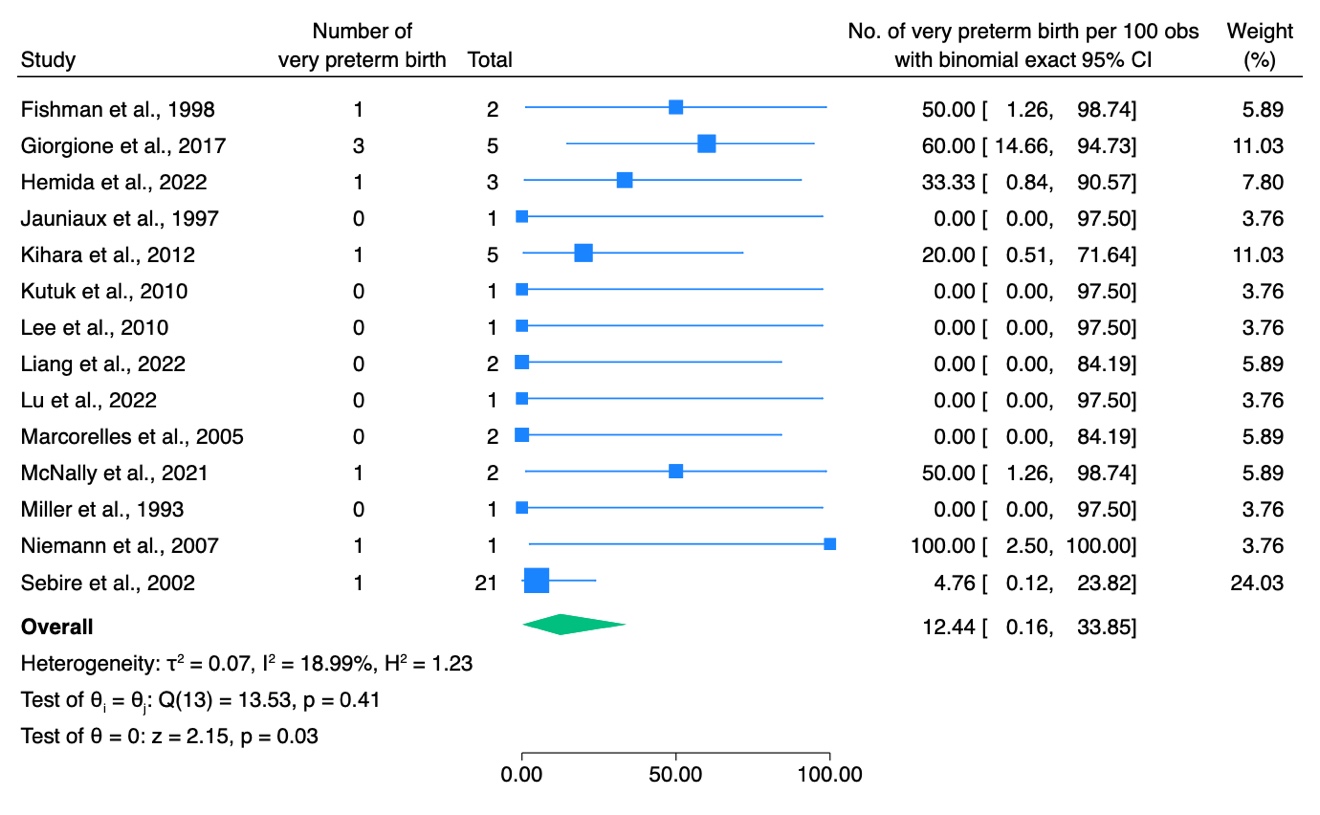


1. mode of delivery: Cesarean


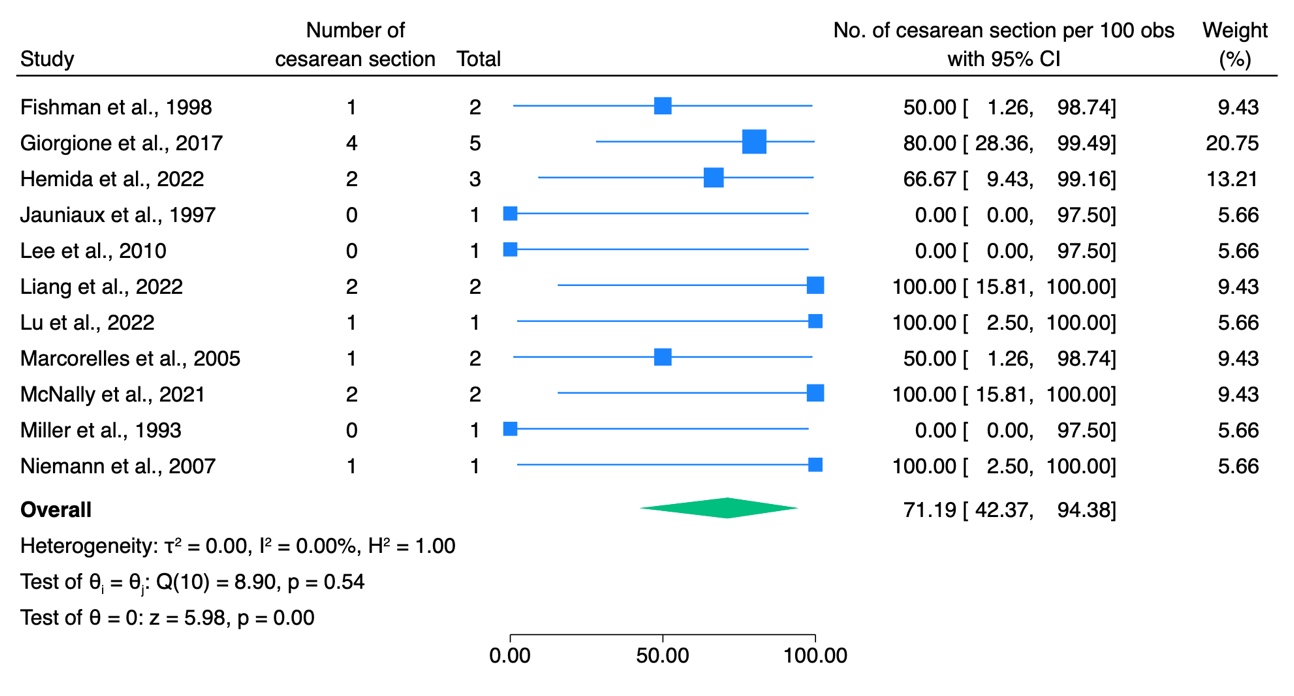


1. mode of delivery: vaginal


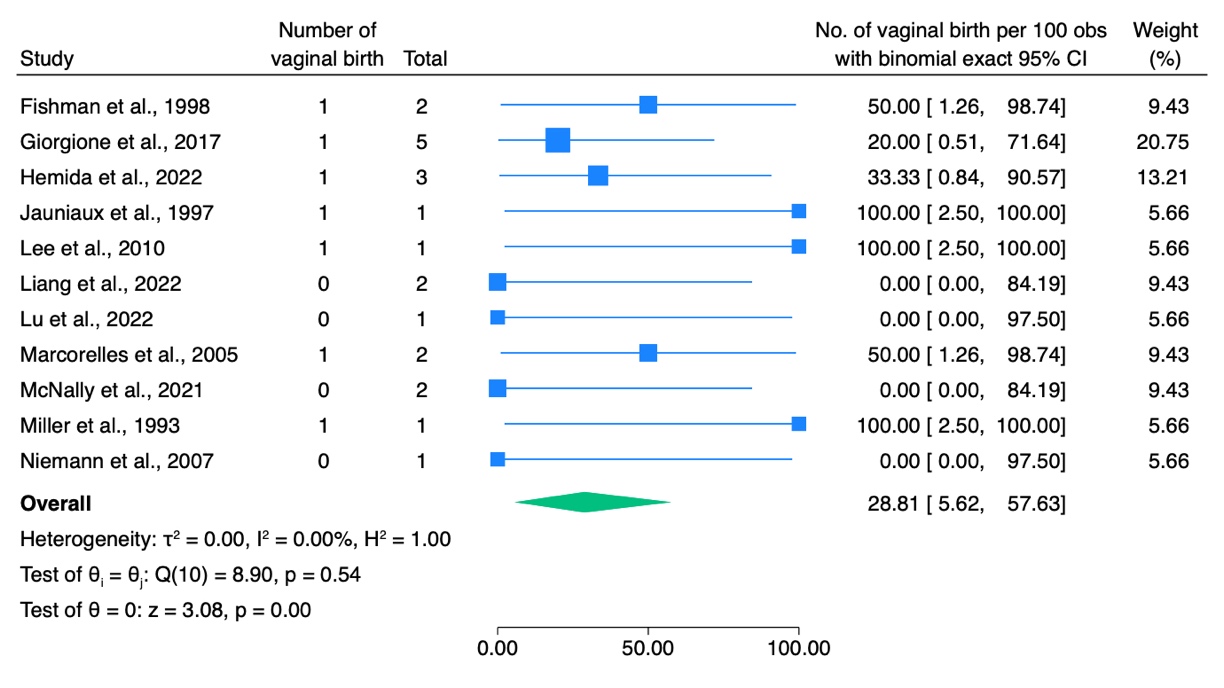


1. mode of delivery: iatrogenic birth


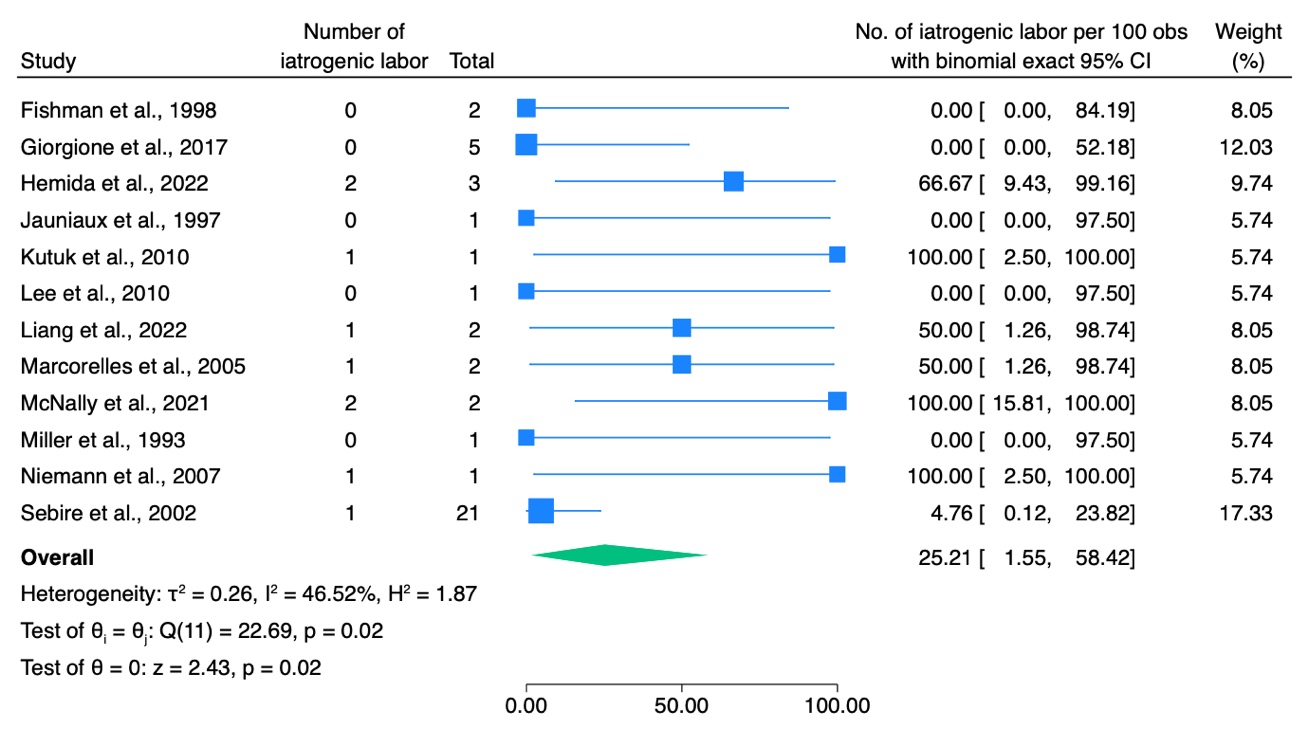


1. neonatal complications: low birth weight (<10°)


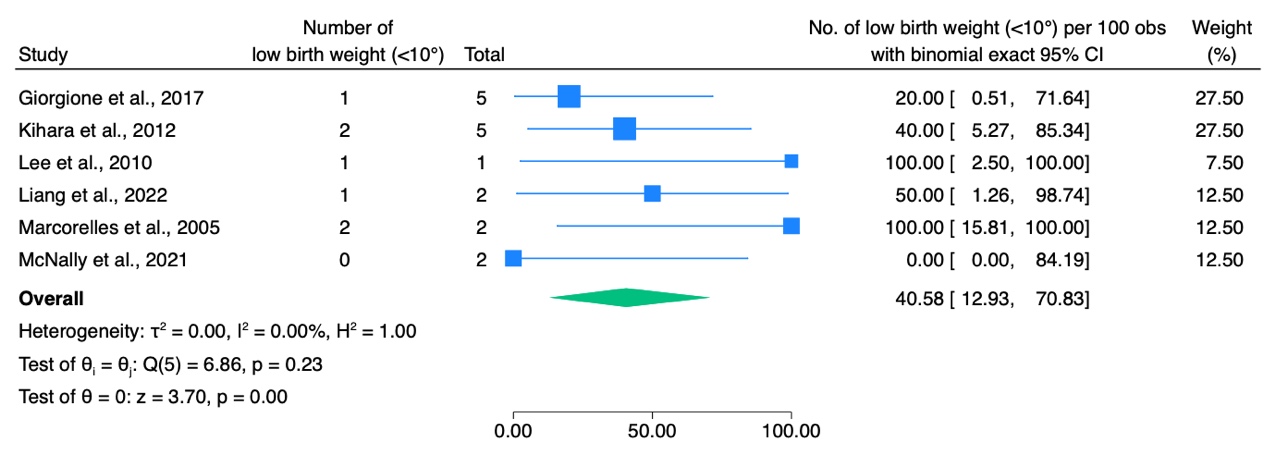


1. neonatal complications: neonatal mortality


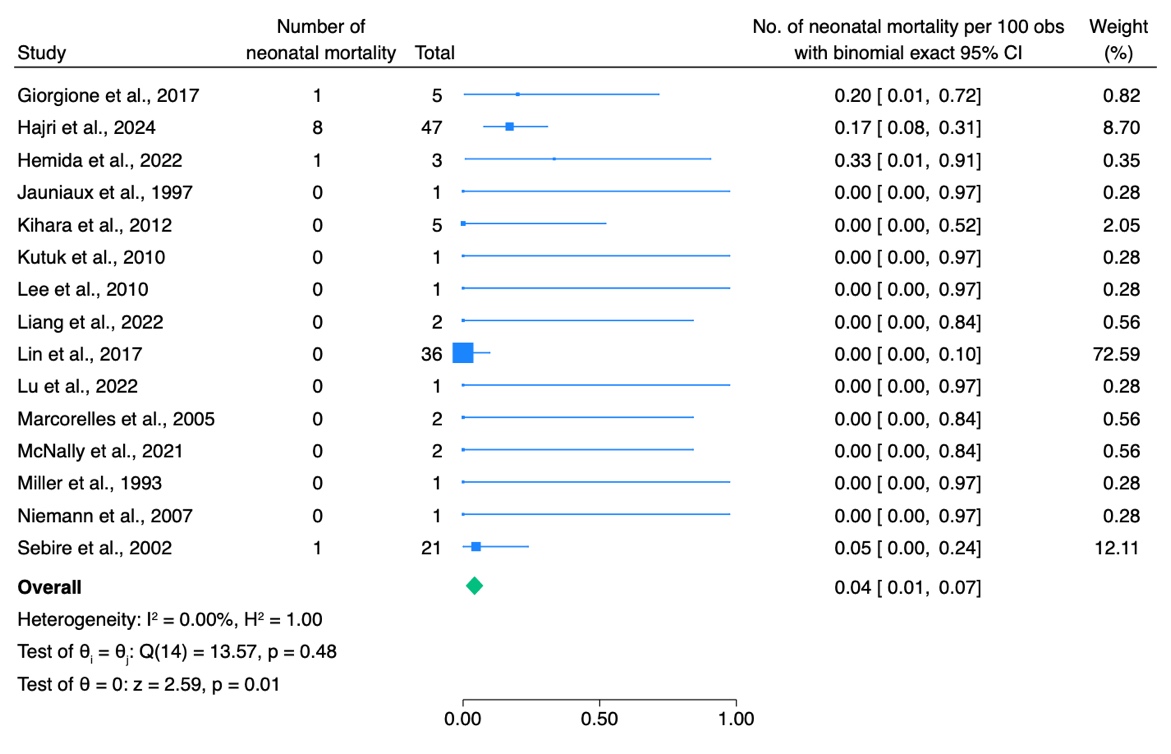


1. maternal complications (peri-partum): post-partum haemorrhage


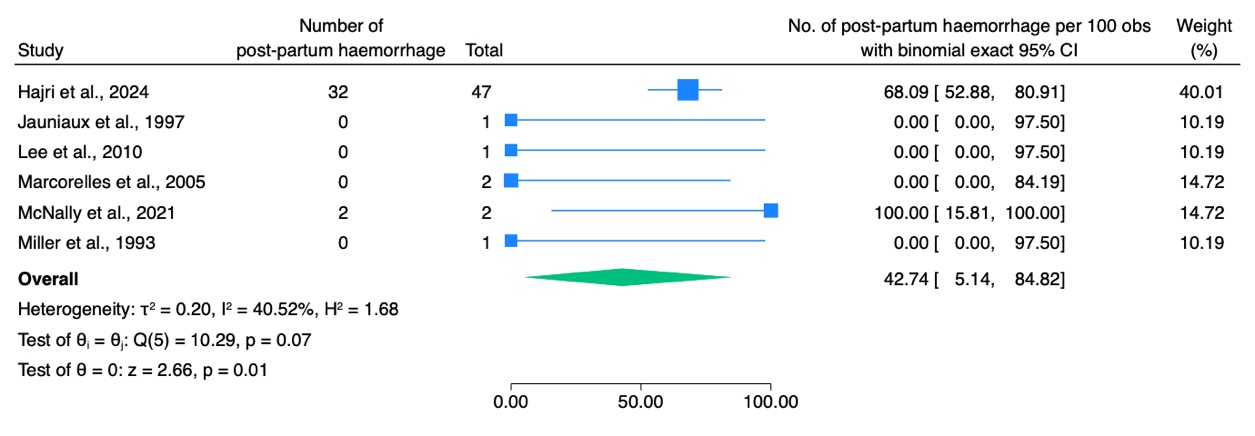


1. maternal complications (peri-partum): maternal mortality


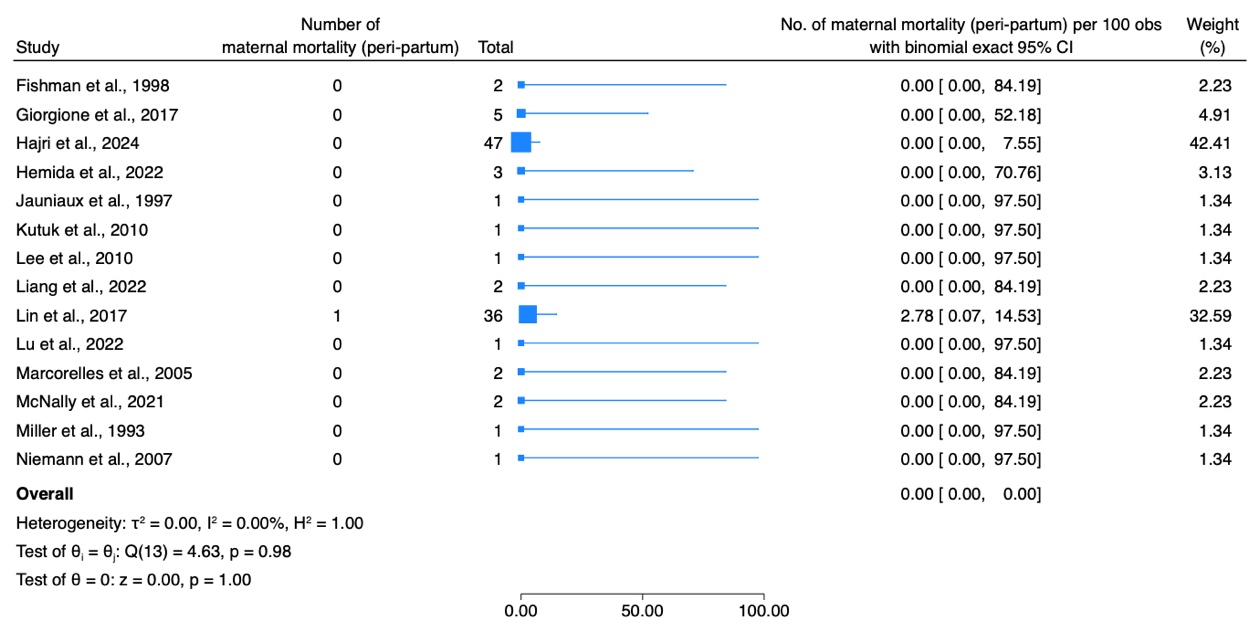


1. GTN (total)


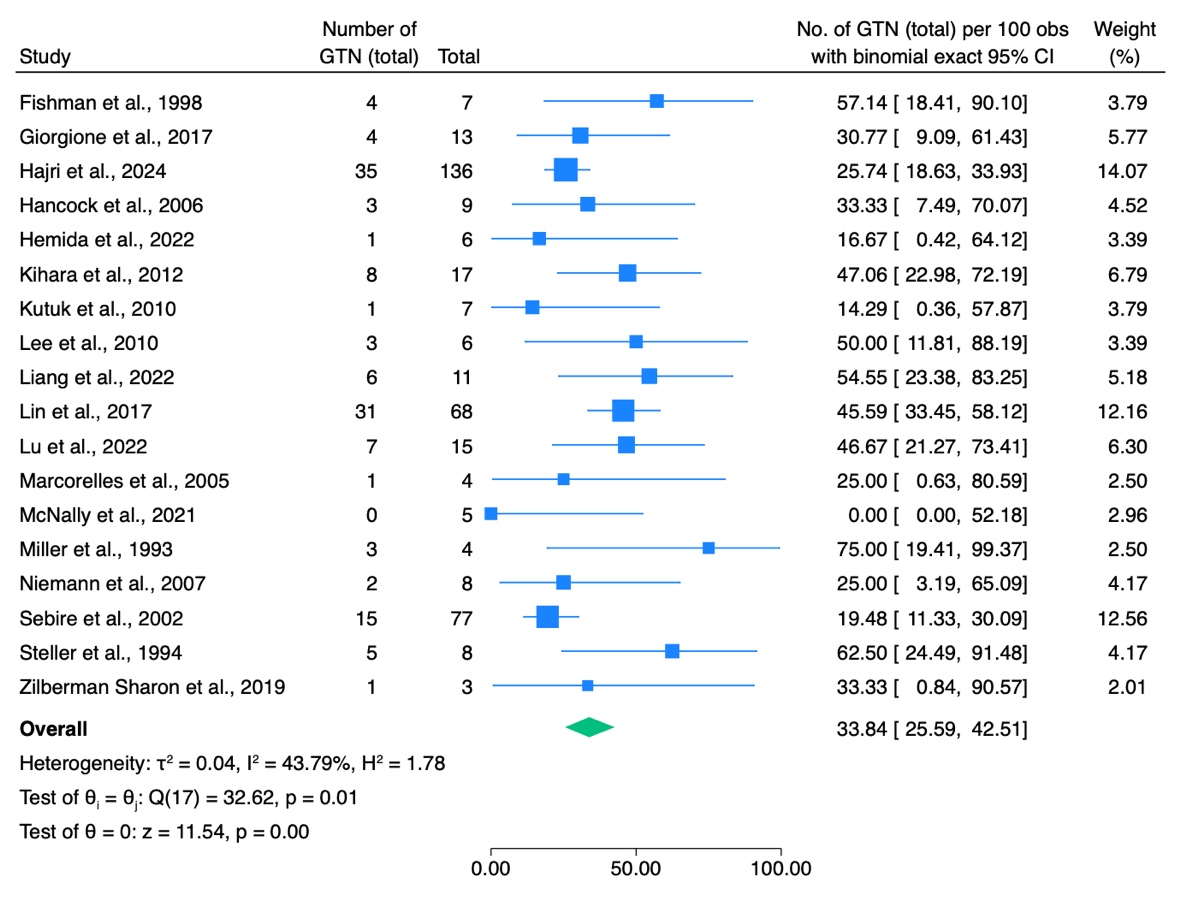


1. GTN in elective TOP


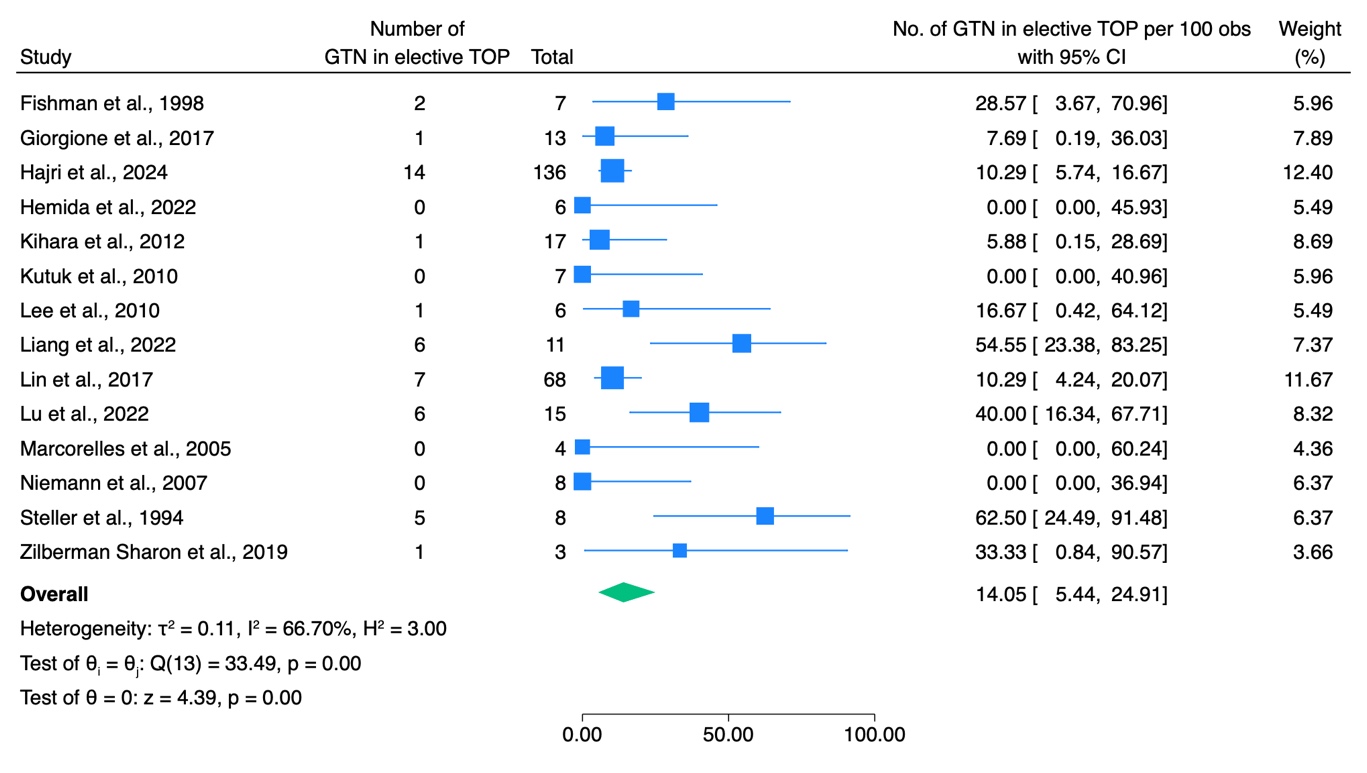


1. GTN in continued pregnancies


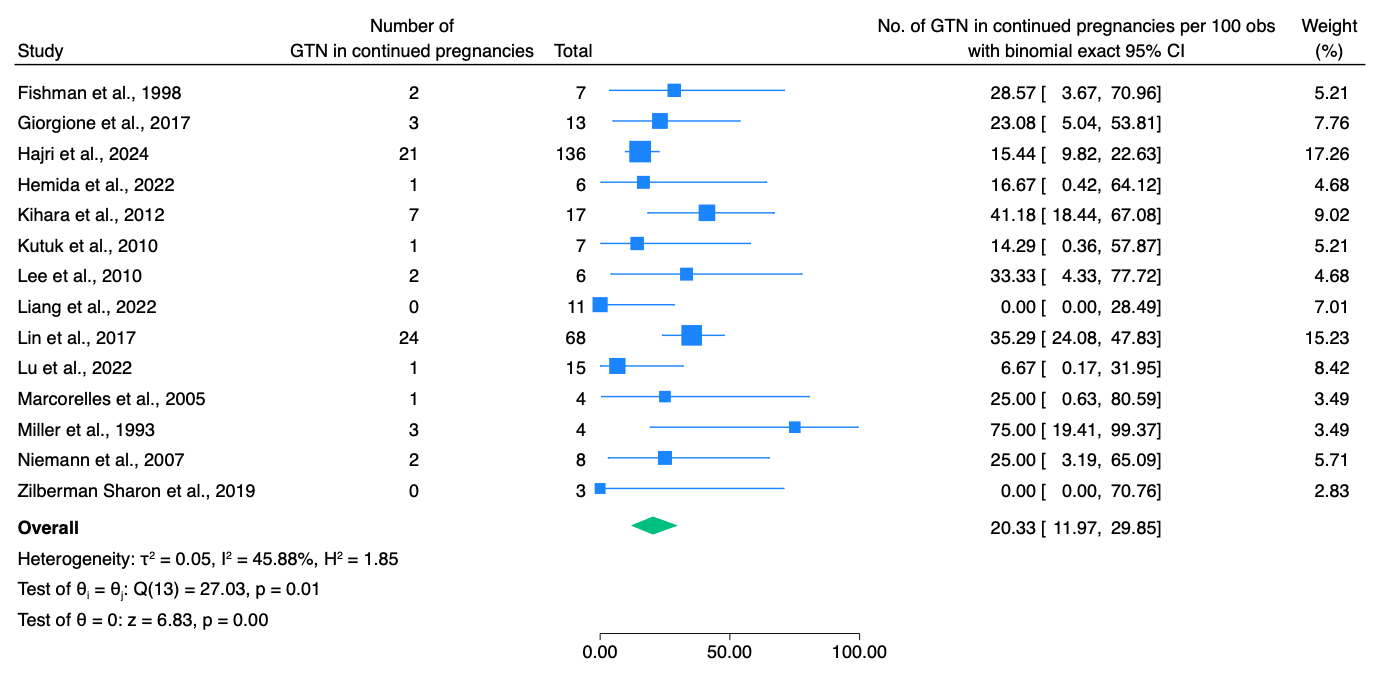


1. GTN in pregnancies ending with a living baby


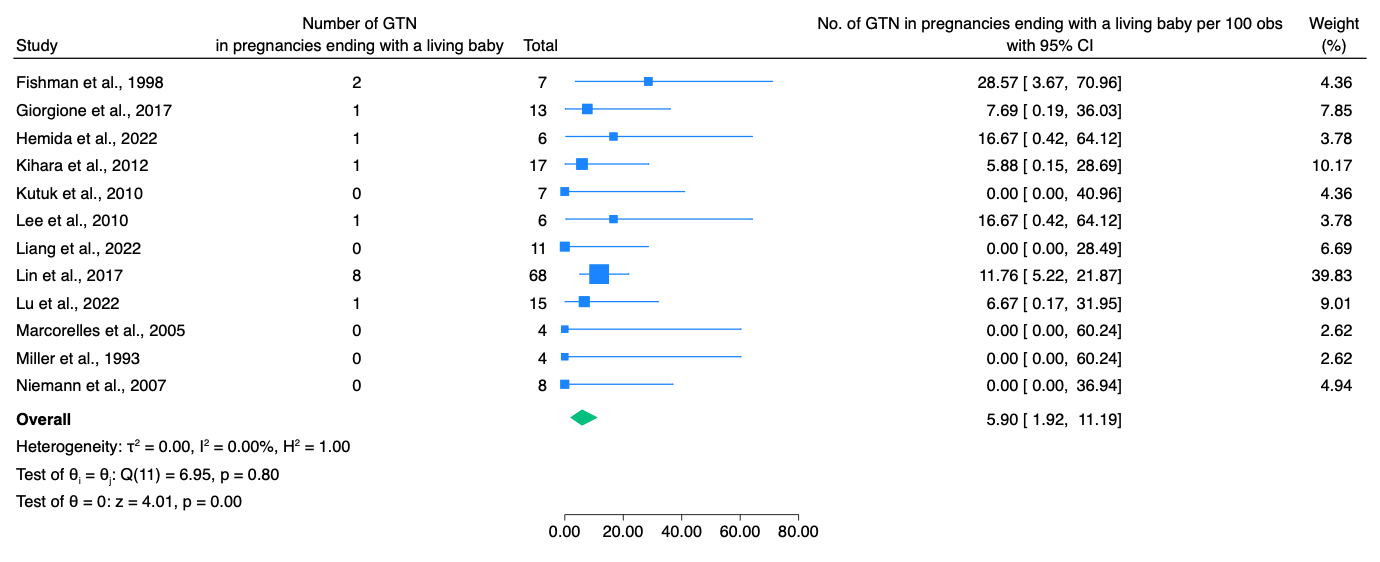

Supplement: Supplementary file 3 — Figure S1 Forest plots of pooled proportions of obstetric and oncological outcomes in complete hydatidiform mole and coexisting normal fetus. [file UOG-67-272-s002.docx]
